# Evolution of mitochondrial genomes in Baikalian amphipods

**DOI:** 10.1186/s12864-016-3357-z

**Published:** 2016-12-28

**Authors:** Elena V. Romanova, Vladimir V. Aleoshin, Ravil M. Kamaltynov, Kirill V. Mikhailov, Maria D. Logacheva, Elena A. Sirotinina, Alexander Yu. Gornov, Anton S. Anikin, Dmitry Yu. Sherbakov

**Affiliations:** 1 Laboratory of Molecular Systematics, Limnological Institute, Siberian Branch of Russian Academy of Sciences, Irkutsk, 664033 Russian Federation; 2 Belozersky Institute for Physicochemical Biology, Lomonosov Moscow State University, Moscow, 119991 Russian Federation; 3 Institute for Information Transmission Problems, Russian Academy of Sciences, Moscow, 127994 Russian Federation; 4 Extreme Biology Laboratory, Institute of Fundamental Medicine and Biology, Kazan Federal University, Kazan, 420012 Russian Federation; 5 Institute for System Dynamics and Control Theory, Siberian Branch of Russian Academy of Sciences, Irkutsk, 664033 Russian Federation; 6 Faculty of Biology and Soil Studies, Irkutsk State University, Irkutsk, 664003 Russian Federation

## Abstract

**Background:**

Amphipods (Crustacea) of Lake Baikal are a very numerous and diverse group of invertebrates generally believed to have originated by adaptive radiation. The evolutionary history and phylogenetic relationships in Baikalian amphipods still remain poorly understood. Sequencing of mitochondrial genomes is a relatively feasible way for obtaining a set of gene sequences suitable for robust phylogenetic inferences. The architecture of mitochondrial genomes also may provide additional information on the mechanisms of evolution of amphipods in Lake Baikal.

**Results:**

Three complete and four nearly complete mitochondrial genomes of Baikalian amphipods were obtained by high-throughput sequencing using the Illumina platform. A phylogenetic inference based on the nucleotide sequences of all mitochondrial protein coding genes revealed the Baikalian species to be a monophyletic group relative to the nearest non-Baikalian species with a completely sequenced mitochondrial genome - *Gammarus duebeni*. The phylogeny of Baikalian amphipods also suggests that the shallow-water species *Eulimnogammarus* has likely evolved from a deep-water ancestor, however many other species have to be added to the analysis to test this hypothesis.

The gene order in all mitochondrial genomes of studied Baikalian amphipods differs from the pancrustacean ground pattern. Mitochondrial genomes of four species possess 23 tRNA genes, and in three genomes the extra tRNA gene copies have likely undergone remolding. Widely varying lengths of putative control regions and other intergenic spacers are typical for the mitochondrial genomes of Baikalian amphipods.

**Conclusions:**

The mitochondrial genomes of Baikalian amphipods display varying organization suggesting an intense rearrangement process during their evolution. Comparison of complete mitochondrial genomes is a potent approach for studying the amphipod evolution in Lake Baikal.

**Electronic supplementary material:**

The online version of this article (doi:10.1186/s12864-016-3357-z) contains supplementary material, which is available to authorized users.

## Background

Ancient freshwater lakes are the birthplaces of very diverse and mostly endemic biota. Their eco-systems are «natural laboratories of evolution» that offer insights into many evolutionary topics, attracting continuous attention of the scientific community and advancing the elucidation of the speciation mechanisms [1, 2]. About ten of the contemporary freshwater lakes existed for longer than one million years. Among them, Lake Baikal is the oldest (reviewed in [1–4]). The age of Lake Baikal is estimated by different authors to be in the range of 25 to 30 million years [5, 6].

Similarly to other ancient giant lakes, a greater part of the huge diversity of animals inhabiting Lake Baikal belongs to the species flocks [7]. These diverse groups of monophyletic species are believed to evolve in the confines of the lake through adaptive radiation in sympatry [2, 8, 9], although geographic isolation is possible in some cases [10–12].

Amphipods are the most diverse group of Baikalian invertebrates (more than 350 described species). They are extremely diverse morphologically and have a wide range of ecological specificities [13, 14].

Most of Baikalian amphipod species have evolved in the confines of Lake Baikal, although some of them have later spread to other water bodies in Eurasia [13, 14]. The only Holarctic species inhabiting shallow bays of Lake Baikal is *Gammarus lacustris* Sars, 1864 [14, 15]. Also *Gammarus dabanus* Takhteev et Mekhanikova, 2000, an endemic species from the mountain streams of Khamar-Daban ridge, was found at the edge of Baikal near the estuaries [16]. The discovery and description of all amphipod species from Lake Baikal is still far from approaching completion (i.e. [12, 16–19]). The work on the revision of their higher level taxonomy is still in progress [13, 14, 17, 19–27]. According to the most modern revision by Kamaltynov [17] all Baikalian amphipod species belong to 76 genera and eleven families, ten of which are autochthonous: Carinogammaridae Tachteev, 2000, Crypturopodidae Kamaltynov, 2001, Macrohectopodidae Sowinsky, 1915, Micruropodidae Kamaltynov, 1999, Baikalogammaridae Kamaltynov, 2001, Ommatogammaridae Kamaltynov, 2009, Acanthogammaridae Garjajeff, 1901, Eulimnogammaridae Kamaltynov, 1999, Pachyschesidae Kamaltynov, 1999, Pallaseidae Tachteev, 2001.

Application of molecular phylogenetic approaches allowed to refine the taxonomy of Baikalian amphipods and to outline the main trends of their evolution, specifically their phylogenetic position relative to the non-Baikalian amphipod taxa. It was shown that at the very beginning of the diversification Baikalian amphipods had split into at least two major lineages delineated by their ecological and morphological traits [28–32]. This observation could also be explained by two independent colonizations of Lake Baikal. Several groups have shown that the ancestors of the extant Baikalian amphipods were closely related to the ancestors of the Holarctic species of *Gammarus* Fabricius, 1775 [28, 30–34].

Englisch et al. (2003) showed that Baikalian species *Parapallasea lagowskii* (Dybowsky, 1874) was «nested within genus *Gammarus*» [33]. In the study by McDonald et al. performed on 32 species of Baikalian amphipods and 29 non-Baikalian species only species belonging to the family Micruropodidae clustered together with *Gammarus* [30]. An early study by Sherbakov et al. based on partial 18S rRNA sequences showed close relationship of the two Baikalian lineages to different species of *Gammarus* [28]. This was later confirmed in the detailed study by Hou and Sket, which utilized more genetic markers and sampled more species of Gammaridae [32]. One lineage of Baikalian amphipods was found to be a sister group to the “Oriental *Gammarus* clade”, the other one was closer to the “Eurasian clade” [32]. Molecular phylogeny also revealed inconsistencies in the current taxonomy of Baikalian amphipods. Genera *Acanthogammarus* Stebbing, 1899 and *Pallasea* Bate, 1862 were shown to be polyphyletic [11, 28, 35].

Majority of the previous molecular studies of evolutionary history and taxonomy of Baikalian amphipods are impeded by insufficient statistical support of the most important clades [28, 30, 35, 36]. The presence of poorly resolved clades might be explained both by insufficient length of sequences used and by their low mutational rate (e.g.18S rDNA) [29, 35]. Unfortunately, more recent works aimed at resolving the complicated relations within *Gammarus* included only a few Baikalian species or did not address their phylogenetic relationship [31, 32, 37, 38]. Therefore, further study of Baikalian amphipods is necessary to elucidate their phylogeny.

In this study we use nucleotide sequences of completely sequenced mitochondrial genomes (mitogenomes) for the evolutionary inferences and clarification of the taxonomy of Baikalian amphipods. The ten studied species appear to form a single clade relative to *Gammarus duebeni* Lilljeborg, 1852. We also perform a detailed comparative structural analysis of amphipods mitochondrial genomes and find an unexpectedly high degree of their length variation and extensive gene rearrangements.

## Results and Discussion

### Mitochondrial genomes organization

We obtained three complete and four nearly complete sequences of mitochondrial genomes from Baikalian amphipod species. Information about animals sampling sites, sequences lengths, numbers of reads, GenBank accession numbers, etc. of currently studied Baikalian amphipod species and that of previously published ones is presented in Table 1. *Acanthogammarus victorii* (Dybowsky, 1874) and *Garjajewia cabanisii* (Dybowsky, 1874) have incomplete non-coding regions sequences due to their low complexity and the presence of repeats (the 51-mer tandem repeats in the control region (CR) of *A. victorii* and the CR sequence duplication in the mitochondrial genome of *G. cabanisii*) that have prevented automatic assembly process. Mitochondrial genomes of *Crypturopus tuberculatus* (Dybowsky, 1874) and *Linevichella vortex* (Dybowsky, 1874) also lack various parts of the coding sequences due to the difficulties in amplification of these regions. The sizes of complete mitochondrial genomes range from 14,370 to 18,114 b.p., which is within the range of mitogenomes of other amphipods (14,113 to 18,424 b.p.). The AT content varies from 62.24 to 68.96% for completely sequenced mitochondrial genomes and from 57.45 to 64.86% for partially sequenced ones (Table 2). In general this is compatible with the values of other known complete amphipod mitogenomes (64.03–76.03%), although the AT content of *Brachyuropus grewingkii* (62.24%) was found to be the lowest within amphipods [39–47].

**Table 1:** Summary of the Baikalian amphipod data presented in the study

Species	Sampling site, coordinates	Ecological features: depth of habitat, armament, nutrition	DNA template type, number of paired reads	Mean coverage of mitogenome	Mitogenome size, b.p.	AT-skew	GC-skew	GenBank nos.	References
*Acanthogammarus victorii*	Listvyanka, 51°51’30” N, 104°50’37” E	1.5–90 m., spiny, predator/scavanger	total DNA, 6.3 M	40×	**17,424** ^a^	0.027	−0.251	KX341962	This study
*Brachyuropus grewingkii*	Estuary of Buguldeyka river, 52°28’N, 106°06’E–52°28’N, 106°05’E	100–1380 m., (usually deeper than 300 m.) spiny, predator	total DNA, 6.4 M	12×	17,118	0.003	−0.307	KP161875	[105]
*Crypturopus tuberculatus*	Estuary of Anga river, 52°46’40” N, 106°34’60” E	1.5–99 m., tuberous, detritophagous	mitochondrial DNA amplicons, 59,961	901×	**13,864** ^b^	−0.070	−0.013	KX341963	This study
*Eulimnogammarus cyaneus*	Sukhoi Ruchey, 51°38’48” N, 103°45’14” E	0–3.5 m., smooth, phytophagous	mitochondrial DNA amplicons, 541,283	7665×	14,370	−0.019	−0.251	KX341964	This study
*Eulimnogammarus verrucosus*	Bol’shie Koty, 51°54’11.67” N, 105°04’07.61” E	0–12 m., (usually 0–3 m.) smooth, phytophagous	total DNA, 352.7 M	1000×	15,314	−0.007	−0.238	KF690638	[71]
*Eulimnogammarus vittatus*	Sukhoi Ruchey, 51°38’ 48” N, 103°45’14” E	0–30 m., (usually 0–3 m.) smooth, phytophagous	total DNA, 4.6 M	21×	15,534	−0.014	−0.222	KM287572	[104]
*Garjajewia cabanisii*	Estuary of Buguldeyka river, 52°28’N, 106°06’E–52°28’N, 106°05’E	80–1250 m., (usually deeper than 200 m.) spiny, predator	total DNA, 7.5 M	24×	**17,576** ^a^	−0.002	−0.286	KX341965	This study
*Gmelinoides fasciatus*	Sukhoi Ruchey, 51°38’ 31” N, 103°46’ 03” E	0–192 m. (usually 0–5 m.), tuberous, phytophagous/ euryphagous	total DNA, 7.4 M	14×	18,114	−0.001	−0.303	KX341966	This study
*Linevichella vortex*	Listvyanka, 51°51’12” N, 104°51’48” E	0–88 m., (usually 1–2 m.) smooth, phytophagous	mitochondrial DNA amplicons, 13,319	132×	**11,444** ^b^	−0.027	−0.223	KX341967	This study
*Pallaseopsis kesslerii*	Estuary of Anga river, 52°46’40” N, 106°34’60” E	1–61 m., slightly spiny, phytophagous	mitochondrial DNA amplicons, 14,144	154×	15,759	0.010	−0.182	KX341968	This study

Bold numbers indicate incomplete sequences

^a^a mitochondrial genome with an incomplete non-coding part

^b^a mitochondrial genome with incomplete sequences of both coding and non-coding parts

*M* denotes million reads

**Table 2:** Comparison of mitogenomic characteristics of Baikalian amphipods

Species	Genome		PCGs		rRNAs		tRNAs		Putative CRs		Intergenic spacers	
	Length, b.p.	AT %	Length, b.p.	AT %	Length, b.p.	AT %	Length, b.p.	AT %	Length, b.p.	AT %	Length, b.p.	AT %
*Acanthogammarus victorii*	**17,424**	59.28	11,043	57.01	1616	63.68	1360	64.34	**1390**	70.86	2035	56.86
*Brachyuropus grewingkii*	17,118	62.24	11,056	60.20	1608	66.36	1304	65.41	1264	73.26	1852	61.56
*Crypturopus tuberculatus*	**13,864**	57.45	**10,803**	56.53	**798**	63.28	**1078**	62.15	-	-	**1207**	57.91
*Eulimnogammarus cyaneus*	14,370	67.59	11,047	66.78	1607	71.81	1300	66.69	181	77.90	268	75.37
*Eulimnogammarus verrucosus*	15,314	68.96	11,022	66.63	1602	69.54	1335	67.42	437	83.30	872	76.63
*Eulimnogammarus vittatus*	15,534	67.42	11,050	65.59	1606	71.30	1373	67.30	1053	79.01	491	72.51
*Garjajewia cabanisii*	**17,576**	64.86	11,052	62.67	1605	66.98	1367	65.47	1212	76.07	**2324**	67.60
*Gmelinoides fasciatus*	18,114	65.87	11,448	63.29	1594	69.01	1348	66.47	235	81.28	3863	70.88
*Linevichella vortex*	**11,444**	64.51	**9813**	64.15	**538**	67.84	**829**	64.29	-	-	**296**	72.97
*Pallaseopsis kesslerii*	15,759	63.10	11,035	61.13	1597	64.87	1361	67.52	340	80.59	1800	70.67

Bold numbers indicate incomplete sequences

All completely sequenced mitochondrial genomes of Baikalian amphipods have strong negative values of GC-skew (prevalence of C over G) on the (+) strand (−0.30 to −0.18) and their AT-skew (prevalence of A over T) varies from −0.02 to 0.01 (Table 1, Additional files 1 and 2). Species with partial mitochondrial genome sequences have GC-skew from −0.25 to −0.01 and AT-skew from −0.07 to +0.02. The unequal nucleotide content between two strands is typical for mitochondrial DNA. This is a consequence of an asymmetric mutational process that affects the A and C nucleotides during the replication and transcription [48, 49].

**Fig. 1:**
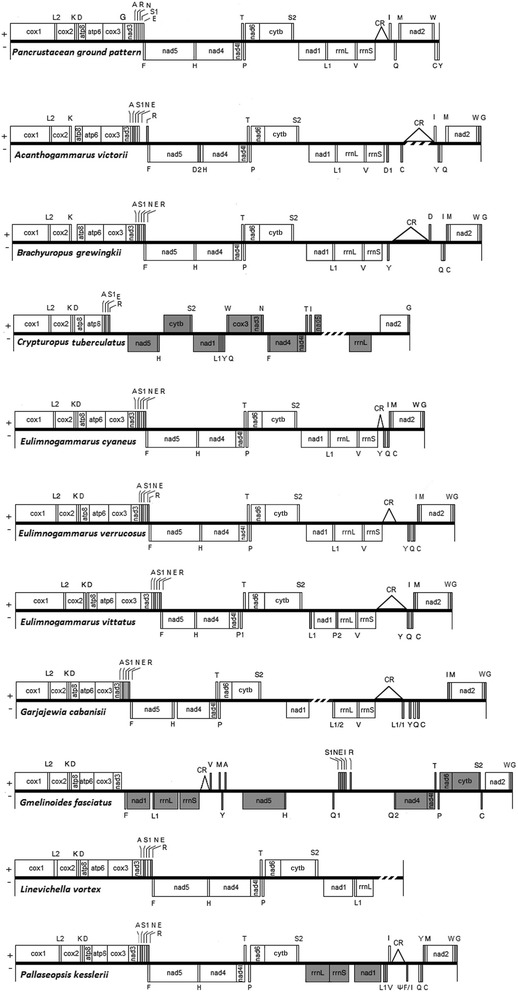
Organization of mitochondrial genomes of Baikalian amphipods in comparison to the pancrustacean ground pattern. Genes on the (+) strand are located above the line, whereas genes coded on the (−) strand are below the line. Transfer RNAs genes are labeled by their single-letter amino acid code. Oblique strokes in some genomes denote unknown areas that were not sequenced due to technical difficulties. Gene features with altered location in comparison to the pancrustacean ground pattern are shown in grey color. Location of a putative CR sequence of *B.grewingkii* has been changed from the one inferred in the previously published article by Romanova et al. [105]

Complete sequences of Baikalian amphipod mitochondrial genomes contain 13 protein coding genes, two rRNA genes and from 22 to 23 tRNA genes, a CR and intergenic spacers of different number and lengths (Fig. 1, annotations of studied mitochondrial genomes are in Additional file 3).

### Protein-coding genes order

All completely sequenced Baikalian amphipod mitochondrial genomes possess a typical set of 13 protein-coding genes (PCGs). PCGs of each species have the same transcriptional polarity as in the pancrustacean ground pattern [50] (Fig. 1), however the order of PCGs has been altered relative to the ground pattern in the mitochondrial genomes of three species under study. *Pallaseopsis kesslerii* (Dybowsky, 1874) has a translocation of the *nad1* gene, *C. tuberculatus* possesses translocation of *nad5, cytB, nad1, cox3, nad3, nad4, nad4l, nad6, Gmelinoides fasciatus* (Stebbing, 1899) has translocations of *nad1, nad5, nad4, nad4l, nad6* and *cytB* (Fig. 1). These re arrangements have never been observed in other amphipod species.

The only feature in common among the amphipods with the altered gene order is that they are relatively shallow water dwellers (Table 1) belonging to the *Micruropus* flock [35]. However, there are other shallow water amphipods in the dataset which have an unaltered order of mitochondrial genes.

In the non-Baikalian amphipods one may also find deviations from the pancrustacean ground pattern. *Onisimus nanseni* G.O. Sars, 1900 has *nad6* and c*ytB* translocation, in some species of *Metacrangonyx* Chevreux, 1909 *cytB* is inverted [39, 40, 45], the species of *Caprella* Lamarck, 1801 have *nad4, nad4l, nad6* translocations and *nad5* inversion. Gene order in the species belonging to *Pseudoniphargus* Chevreux, 1901 differs from the pancrustacean ground pattern and is variable within the genus. *P. daviui* Jaume, 1991 has *nad1* translocation and *P.sorbasiensis* Notenboom, 1987 has *nad6,* c*ytB* translocation.

Non-Baikalian amphipods with rearranged PCGs relative to the pancrustacean ground pattern are distant from each other taxonomically (up to a superfamily level) as well as ecologically. Among the species with an altered gene order there are two genera of freshwater stygobionts: Palearctic genus *Metacrangonyx* (superfamily Hadzioidea) and Nearctic genus *Pseudoniphargus* (superfamily Allocrangonyctoidea) [34], Arctic marine species *O.nanseni* (superfamily Lysianassoidea) [42] and some species of *Caprella* (superfamily Caprelloidea) inhabiting warm seas around the world [51, 52]. Still, amphipods carrying the unchanged ground pattern are at least equally taxonomically and ecologically diverse. *Parhyale hawaiiensis* (Dana, 1853) (superfamily Talitroidea) is a marine species distributed in north Atlantic [53], *Bahadzia jaraguensis* Jaume and Wagner, 1998 (superfamily Hadzioidea) is a stygobiontic species that was found in the caves of West Indian and Caribbean regions [54, 55], *G. duebeni* (superfamily Gammaroidea) is a North Atlantic distributed species [56], *Gondogeneia antarctica* (Chevreux, 1906) (superfamily Calliopioidea) is a South Antarctic species [57].

It seems that the changes in the gene order of Baikalian as well as non-Baikalian amphipods can not be directly correlated with any meaningful environmental parameter. Similarly, although it is possible that some lineages are more prone to reshuffling of their mitochondrial genes then the others, they do not appear to be phylogenetically clustered. Obviously, further careful investigation with a more comprehensive dataset is necessary to resolve the ancestral pattern of Baikalian amphipod mitochondrial genomes.

### Base composition bias in protein-coding genes

**Fig. 2:**
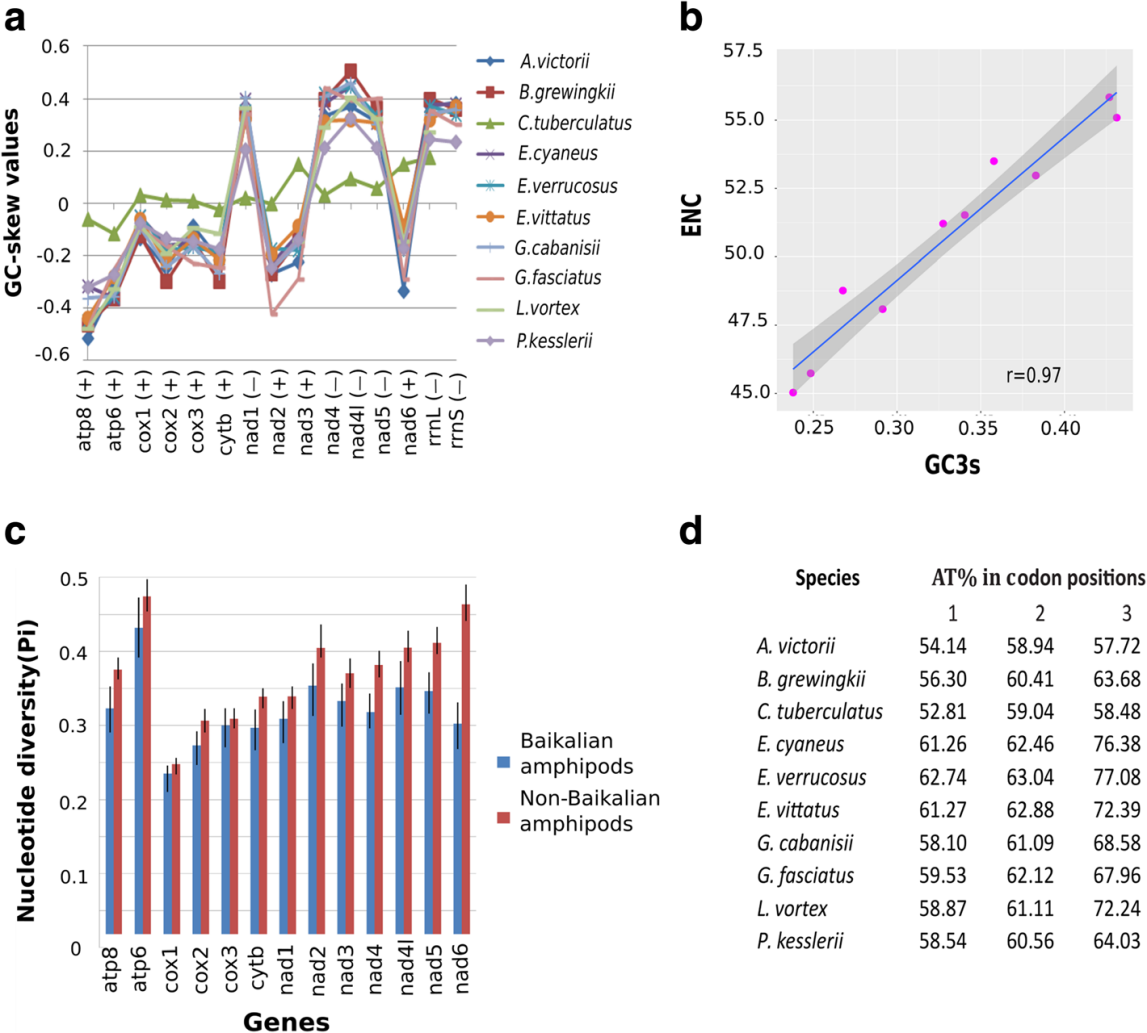
Statistical data for PCGs and ribosomal genes in mitochondrial genomes of Baikalian amphipods. **a** GC-skew values of PCGs and ribosomal genes. (+) marks genes coded by the positive strand, (−) marks genes coded by the negative strand. **b** An illustration of a positive correlation between the effective number of codons (ENC) and the GC content at the third codon position (GC3s) in Baikalian amphipods. **c** Nucleotide diversity (Pi) values for PCGs of Baikalian and non-Baikalian amphipods. **d** AT content (%) in three codon positions in PCSs of Baikalian amphipods

The GC-skew values were measured for all protein coding genes similarly to the measure of strand composition bias [58]. Mitochondrial genomes of most Baikalian amphipod species studied here have negative GC-skew in PCGs that are located on the (+) strand. PCGs encoded on the (−) strand, on the contrary, have positive GC-skew (Fig. 2, Additional file 2). Such strand bias is typical for most mitochondrial genomes in Malacostraca [42, 59]. However, in *C. tuberculatus*, the composition bias was found to be noticeably lower than in the other species. The GC-skew values of the (+) strand PCGs vary from −0.11 to +0.14 and of the (−) strand PCGs GC-skew values vary from +0.02 to +0.09 (Fig. 2). In the *nad3* and *nad6* genes the strand bias is reversed and thus the GC-skew is positive.

The strand bias reversion was previously detected in the mitochondrial genomes of several different invertebrate species such as *Katharina tunicata* (Mollusca), *Florometra serratissima* (Echinodermata), *Argiope amoena* (Chelicerata), *Aleurochiton aceris, Bemisia tabaci, Campanulotes bidentatus, Thrips imaginis, Bothriometopus macrocnemis, Cotesia vestalis, Neomaskellia andropogonis, Tetraleurodes acacia* ect. (Hexapoda), *Ligia oceanica*, *Hutchinsoniella macracantha, Tigriopus californicus, Lepeophtheirus salmonis, Calanus hyperboreus, Argulus americanus, Procambarus clarkii, Corallianassa couitierei, Nihonotrypaea japonica, Cambaroides similis, Homarus gammarus* ect. (Crustacea) [43, 45, 49, 58]. Within amphipods the reversed strand bias was found in the mitochondrial genomes of Metacrangonyctidae species [45]. As previously suggested by Hassanin et al., 2005, the strand reversion can happen due to the reversion of one or several genes relative to the CR or alternatively, it could be explained by the reversion that includes the CR [49]. We did not succeed in sequencing the CR of *C. tuberculatus* and thus we can not conclude which scenario is more likely in this case. Still, taking into account the relatively low values of GC skew of the majority of protein coding mitochondrial genes in this organism, one may speculate that the reversion is a relatively recent event on the evolutionary time scale [49]. Taxa with a reversed strand bias should be taken into phylogenetic analysis with caution as they tend to promote long branch attraction artifacts [49].

### AT content in protein-coding genes and codon usage

The AT content of completely sequenced PCGs varies from 57.01% (in *A.victorii*) to 66.78% (in *Eulimnogammarus cyaneus* (Dybowsky, 1874)) (Table 2). The PCGs of *A. victorii* have the lowest AT content among entirely sequenced mitochondrial genomes of amphipods. Codon positions in mitochondrial PCGs were noted to have an unequal AT content. Mitochondrial genomes studied here have the lowest AT content at the first codon position, a slightly higher AT content at the second position and the highest AT content at the third codon position (Fig. 2). The *A. victorii* and *C.tuberculatus* species*,* however, have the highest AT content at the second codon position in their PCGs, and the lowest at the first codon position (Fig. 2).

Codon usage analysis in Baikalian amphipods revealed the presence of all codon types typical for the invertebrate mitochondrial code (Additional file 4). Along with the regular start codons (ATA and ATG), the unusual ones (ATT, ATC, TTG and GTG) were predicted to initiate mitochondrial PCGs. Presence of unusual start and stop codons is generally typical for mitochondrial genomes [60]. Some PCGs of the investigated genomes possess truncated stop codons (T) that are presumably completed after a post-transcriptional polyadenylation [61] (Additional file 3).

In all currently studied genomes the most frequently used codons are TTT (Phe) (5.40 to 7.05%) and TTA (Leu) (4.69 to 8.72%). Also some of the more frequent codons include ATT (Ile) (2.93 to 5.54%), ATA (Met) (3.69 to 6.07%) (Additional file 4). In non-Baikalian amphipods these four codons are also among the most abundant ones. Bias toward the AT-rich codons is typical for many arthropods [62].

The Effective Number of Codons (ENC) [63] in Baikalian amphipods varies widely, ranging from 45.04 in *Eulimnogammarus verrucosus* (Gerstfeldt, 1858) to 55.67 in *C.tuberculatus* (Fig. 2). These values exceed slightly the ENC in other amphipod species, which range from 36.47 to 49.74. A strong positive correlation (*r* = 0.97) between ENC and GC content at the third codon position was found in mitochondrial genomes of Baikalian amphipods (Fig. 2). The ENC in all sequenced genomes increases with the decrease of AT content in protein coding genes.

### Nucleotide diversity analysis

Nucleotide diversity (Pi) was estimated for two groups of protein-coding genes, the first one encompasses the Baikalian amphipod species, the second one contains non-Baikalian species (see species list in Additional file 5) (Fig. 2). These data may be useful for designing new molecular markers for phylogenetic inferences of amphipods. The patterns of nucleotide diversity within mitochondrial genes in both groups were nearly identical, with lower values for genes of Baikalian amphipods. This may be due to the much higher evolutionary span of the non-Baikalian amphipod species in comparison to the Baikalian amphipods. Indeed, all Baikalian amphipods belong to a single superfamily Gammaroidea, while the rest of analyzed amphipods represent superfamilies Hadzioidea, Allocrangonyctoidea, Lysianassoidea, Caprelloidea, Talitroidea, Gammaroidea, Calliopioidea.

In both groups the *cox1* sequences have the smallest values of diversity (0.23 ± 0.01 for Baikalian species, and 0.24 ± 0.01 for non-Baikalian ones), whereas the *atp8* appears to be the most variable (0.43 ± 0.03 for Baikalian species and 0.47 ± 0.01 for non-Baikalian ones) (Fig. 2). The highest difference in the nucleotide diversity between Baikalian and non-Baikalian species was noted in the *nad6,* which might be caused by the lack of a complete sequence from *L.vortex.* Other studies dedicated to animal mitochondrial genomes also revealed the *cox* genes to be the most conserved and the *atp* genes to be the most variable [45, 64–68]. However, there are animal groups with the different patterns of nucleotide variability distribution within protein coding genes of their mitochondrial genomes [69, 70]. It seems that the pattern of nucleotide diversity is a lineage specific feature.

On the basis of the data of all available Baikalian amphipod mitochondrial genomes it is possible to predict which genes are more suitable for different phylogenetic applications in this taxonomic group. The most variable genes (*atp8, nad2, nad4l, nad5, nad6*) can be utilized as markers for species level phylogeny and population studies. Alternatively, sequences of the least variable mitochondrial *cox* genes may be suitable for deep phylogeny (families and higher taxa) investigations.

### Transfer RNA genes

From 22 to 23 tRNA genes were identified in the completely sequenced mitogenomes of Baikalian amphipod species. The locations of tRNA genes are highly variable and all studied mitochondrial genomes have tRNA genes with altered positions relative to the pancrustacean ground pattern (Fig. 1).

The secondary structures of 213 mitogenome-encoded tRNAs ranging from 49 to 64 b.p. in length and including those of *E.verrucosus* [71] were predicted for the analysis (Additional file 6). Most of the predicted tRNAs possess the expected clover-leaf structures, however some of them have aberrant structures. The tRNA-Ser (UCU) lacks the DHU arm in all studied species. The DHU arm is also missing in the tRNA-Val of *B.grewingkii, A.victorii, E.cyaneus*, and *G.cabanisii*, in the tRNA-Ser (UGA) of *G.fasciatus* and *L.vortex*, and in the tRNA-Tyr of *G.fasciatus.* The tRNA-Gln lacks the TψC arm in all studied species except for *C.tuberculatus*. The TψC arm is also absent in the tRNA-Thr of *G.fasciatus* and in the tRNA-His and tRNA-Pro of *P.kesslerii* (Additional file 6).

The presence of tRNAs with aberrant structures was found to be typical for crustaceans [39, 41–45, 47, 72–76] and can also be seen in other invertebrates [77, 78]. The presence of aberrant tRNAs in mitochondrial genomes is usually explained either by selection towards minimization of the mitochondrial genome [77] or by replication slippage [79].

In four out of ten studied mitogenomes the additional tRNA gene copies are found along with the standard set of 22 tRNA genes. A copy of *trnP* is found in *Eulimnogammarus vittatus* (Dybowsky, 1874), *trnL1* in *G.cabanisii*, *trnQ* in *G. fasciatus* and *trnD* in *A. victorii* (Fig. 1, Additional files 3 and 6). The copies of tRNA-Pro*,* tRNA-Leu1 and tRNA-Asp possess the standard clover-leaf structure. In *G. fasciatus* a clover-leaf structure is seen in one tRNA-Gln copy (Q2), whereas the other copy (Q1) lacks the TψC arm (Additional file 6). *P.kesslerii* also has an additional tRNA adhering to a typical clover-leaf structure, which however has four bases at the presumed anticodon site. The nucleotides in the middle of the anticodon loop may correspond to either Phe or Ile depending on the exact position of the anticodon. An abnormal anticodon suggests that this copy is a pseudogene (ψ F/I) (Additional file 6).

The presence of additional tRNA genes was noted in many mitochondrial genomes of both vertebrate and invertebrate species. If the duplications occurred recently both copies are either identical or very similar in the primary sequence [80–83]. The copies maintain functionality if they retain some conserved elements of their secondary structure [84]. One of the two copies may subsequently undergo elimination. The vestiges of such copies that accumulated substitutions are identified as pseudogenes [85, 86]. Another possible mechanism involves the switch of tRNA identity as the consequence of a point mutation in the anticodon. This mechanism was called tRNA remolding by Cantatore et al. [87].

While the *trnL1* genes of *G.cabanisii* share 75.00% identity pointing to their origin from a single *trnL1* gene, the tRNA duplicates in other amphipods may have switched their identities: one of the two *trnP* genes in *E. vittatus* has 78.68% identity with the *trnL1*, in *G. fasciatus* a copy of *trnQ* has 70.00% identity with the *trnH* gene, and in *A. victorii* a copy of *trnD* gene has 67.21% identity with the *trnH* gene. It is likely that the additional tRNA genes of *E. vittatus, G. fasciatus* and *A. victorii* have been undergone remolding.

Evidence of tRNA remolding was found in mitochondrial genomes of different groups, including Porifera [88–91], Mollusca [92, 93], Echinodermata [87], Arthropoda [43, 91], Chordata [94, 95]. It was recently shown that the remolding event of Trp (UCA) to Gly (UCC) took place in the ancestor of amphipods [91]. Additional tRNA copies found in the mitogenomes of Baikalian amphipods illustrate different stages of the rearrangement process.

### Ribosomal RNA genes

The rRNA genes in mitochondrial genomes of all studied amphipods are located on the (−) strand. In two out of eight mitochondrial genomes with the completely sequenced coding portion (*G.fasciatus* and *P.kesslerii*) the rRNA genes have altered positions in comparison to the pancrustacean ground pattern [50] (Fig. 1, Additional file 3). Rearrangements in the mitochondrial genome involving ribosomal RNA genes or/and PCGs occur much less frequently than those involving only tRNA-coding genes [96–98], thus they are called «major rearrangements» [98]. The rearrangements of rRNA genes and PCGs might potentially affect the effectiveness of replication and transcription of mitochondrial genomes.

The length of completely sequenced Baikalian amphipods’ *rrnL* genes varies from 976 to 984 b.p., and the *rrnS* gene length varies from 618 to 630 b.p. (Additional file 7). The lengths of rRNA genes in Baikalian amphipods appear to be slightly smaller than those in previously sequenced amphipod mitochondrial genomes [40–42, 44–47]. The AT content ranges from 64.50 to 73.75% in the *rrnL* genes, and from 62.38 to 70.90% in the *rrnS* genes respectively (Additional file 7). The GC-skew calculations for rRNA genes give highly positive values (from 0.23 to 0.39 for completely sequenced genes) that are comparable to the values calculated for PCGs encoded on the (−) strand (Additional file 7).

### Control region and intergenic spacers

Mitochondrial genomes of Baikalian amphipods have varying numbers and lengths of non-coding regions (Additional file 3). Control region (CR) is the most important non-coding part involved in replication and transcription of the mitochondrial DNA [99]. For identification of a putative CR we searched features typically associated with such regions in invertebrates (poly-T stretch, tandemly repeated sequences, hairpin structures, AT-rich sequences (Additional file 1) [49, 58, 99–103].

In the mitochondrial genome of *E. cyaneus* a putative CR is a 181 b.p sequence between the *rrnS* gene and the *trnY-trnQ-trnC-trnI-trnM-nad2* gene cluster*.* A similar CR location was also observed in the previously published mitochondrial genomes of *E.vittatus* (1053 b.p.) [104] and *E.verrucosus* (473 b.p.) [71] and also in the mitochondrial genome of *G. duebeni* [44] (Fig. 1, Additional file 3).

Mitochondrial genomes of *A.victorii* and *B.grewingkii* [105] possess large non-coding sequences between the *rrnS* and *nad2* genes. These sequences are interrupted by several tRNA genes. A putative CR in *A.victorii* is located between the locus containing a 13-T stretch and the *trnI* gene. This region was not sequenced completely, however all features typical for a CR were found in the partial sequence of the 1390 b.p. long locus. A putative CR in *B.grewingkii* is a 1264 b.p. long stretch between the 11-T locus and the *trnD* gene (Fig. 1, Additional file 3).

In the mitochondrial genome of *G.cabanisii* the CR is located between the *rrnS* gene and one of the duplicated *trnL1 (trnL1/1)* and measures 1212 b.p. in length. (Fig. 1, Additional file 3). Additionally we found traces of a second CR-like sequence located between the *nad2* gene and the *trnL1/2-rrnL-trnV-rrnS* gene cluster. This region was not sequenced in its entirety, only the regions of 102 b.p. and 232 b.p. corresponding to its flanks were sequenced. The 102 b.p. region has a 98.03% identity with the corresponding region of the original CR, whereas the 232 b.p. region has a 65.60% identity with the other flank of the original CR (Additional file 8). It is likely that the latter region has undergone degeneration after the duplication. Without the complete sequence it is difficult to estimate the time of duplication and the exact sequence of events that led to the appearance of the second CR or speculate about its possible function. Thus we annotated these sequences simply as non-coding regions. The CR duplications were observed in mitochondrial genomes of some invertebrates, including ticks [106, 107], ostracods [108], sea cucumbers [109], katydids etc. [110]. Amphipods *Caprella mutica* Schurin, 1935 and *Caprella scaura* Templeton, 1836 also possess highly identical duplicated CRs in their mitochondrial genomes [41, 43].

The position of CR between the *rrnS* and *nad2* genes in the mitochondrial genomes is typical for some other amphipods: *G. duebeni* [44], *O. nanseni* [42], *G. antarctica* [46], several species of *Pseudoniphargus* [47] and for the pancrustacean ground pattern as well [50], although the adjacent tRNA genes are often different.

The mitochondrial genome of *P.kesslerii* has a large non-coding sequence between the *nad1* and *nad2* genes that is separated by a few tRNA genes and one pseudo tRNA gene. A 340 b.p. sequence located between the 10-T locus and the pseudo tRNA gene (ψ F/I) was defined as a CR (Fig. 1, Additional file 3).

The mitochondrial genome of *G. fasciatus* possesses two large non-coding regions: one between the *rrnS* and *nad5* genes, the other between the *nad5* and *nad4* genes. Both of these regions are interrupted with several tRNA genes. A 235 b.p. region between the *rrnS* and *trnV* was identified as a CR. All other non-coding regions were annotated as intergenic spacers (Fig. 1, Additional file 3).

It is notable that while most features typical for the CRs are found in the corresponding regions in the majority of sequenced mitochondrial genomes of Baikalian amphipods, the mitogenomes of *E. cyaneus* and *A.victorii* only possess tandem repeats.

Variation in the CR location was found in other amphipod species with an altered protein-coding and ribosomal RNA gene order. In the mitochondrial genomes of species from the *Metacrangonyx* genus the CR sequences were identified between the *rrnS* and *cytB* genes [39, 40, 45]. In *C. mutica* and *C. scaura* [41, 43] the first CR is located between the *nad6* gene and the *trnC-cytB* gene cluster, and the second CR is located between the *nad4l-trnP* and the *trnI-trnM-trnY-trnQ-nad2* gene clusters. The lengths of CR sequences in non-Baikalian amphipods also vary significantly from 25 b.p. in *M. goulmimensis* to 1626 b.p. in *G. duebeni* [40–42, 44–47]. Furthermore, it was previously shown that this feature varies considerably even between individuals of the same species. Such CR length variation was noted in *Metacrangonyx longipes* Chevreux, 1909 (26 and 40 b.p.) and *Metacrangonyx goulmimensis* Messouli, Boutin and Coineau, 1991 (25 and 471 b.p.) [40, 45]. Thus, the variable location and length of CR sequences in mitochondrial genomes of the Baikalian amphipod species is in concordance with the characteristics of CRs in other amphipods and invertebrates in general.

The number of intergenic spacers in completely sequenced mitochondrial genomes of Baikalian amphipods varies from 9 to 21 and their total length varies from 268 to 3863 b.p. (Table 2, Additional file 3). Three Baikalian amphipods with entirely sequenced mitochondrial genomes (*B.grewingkii*, *P.kesslerii*, and *G.fasciatus*) possess the largest portions of intergenic spacers which take up 10.80, 11.42, and 21.32% of their complete mitochondrial genomes length. Within the currently sequenced non-Baikalian amphipods only *G. antarctica* [46] has a comparable length of the non-coding intergenic spacers (4354 b.p. in total). The presence of large and numerous intergenic spacers in some mitochondrial genomes studied here may be an evidence of former duplication events [111].

### Distinct features of Baikalian amphipod mitochondrial genomes

Structural analysis of Baikalian amphipod mitochondrial genomes identified several features that differ in most of the studied mitogenomes. Alterations in gene order, strand bias reversion, presence of additional tRNA genes and large non-coding regions as well as a CR duplication indicate intense rearrangement processes, which have occurred during the evolution of Baikalian amphipods. Such rearrangements are usually a result of two major mechanisms, i.e. duplication and subsequent loss of mitochondrial genome regions [112, 113] and intramitochondrial recombination [114]. It is likely that different number and type of rearrangements led to the observed patterns of gene order and variance in the non-coding regions in the mitochondrial genomes of Baikalian amphipods. To decipher the mechanisms behind the appearance of every pattern and to predict an ancestral pattern for the mitogenomes of Baikalian amphipods a further careful investigation with a more comprehensive dataset is necessary.

### Phylogenetic inference

For morphological identification of Baikalian amphipod species in our study we used the most modern taxonomy [17]. This taxonomy become a result of successive revision of previous ones [13, 20–22, 24, 26, 27]. Since new families were introduced in the course of recent revisions of Baikalian amphipods, some species and genera were renamed and thus the species list differs from the one given in the previous publications [28, 30, 35, 36]. To make the phylogenetic results of our work comparable with previous studies we considered them in the context of contemporary taxonomic system that combined all taxonomy alterations [17].

**Fig. 3:**
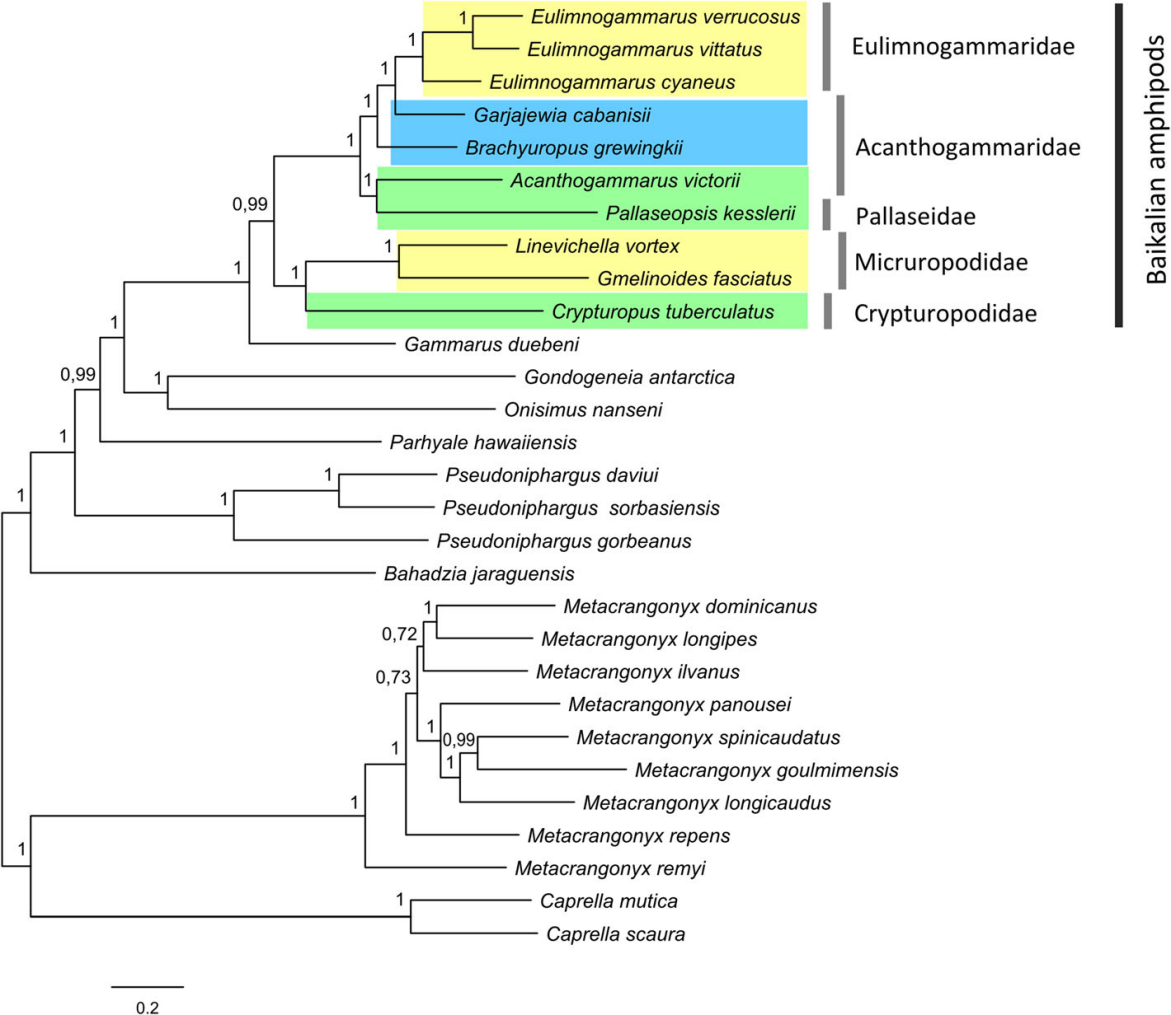
Phylogenetic inference (BI) of amphipods based on 13 mitochondrial protein-coding genes sequences. Numbers above the branches indicate Bayesian posterior probabilities. *Coloured rectangles* denote depths of habitats of Baikalian amphipods. *Yellow rectangles* denote littoral species, *green* ones denote sub-littoral species, *blue* ones denote abyssal species

The phylogenetic analyses of amphipod species based on 13 concatenated mitochondrial protein coding gene sequences using Bayesian Inference (BI) resulted in a well-supported tree. The Baikalian species in the BI tree form a monophyletic group and the amphi-Atlantic amphipod species *G. duebeni* is the nearest outgroup to the Baikalian clade (Fig. 3).

The previous phylogenetic inferences based on the *cox3* gene fragment [36], 18S rRNA and the *cox1* gene fragments [28, 35] as well as on a combined set of molecular data (*rrnL* and 18S rRNA, *cox1* genes fragments) and morphological characters [30] and the most recent study, which involved four molecular markers (*cox1, EF-1α*, 18S and 28S rRNA) [32], revealed that different species of a genus *Gammarus* were included into a clade of Micruropodidae family. The phylogenetic trees derived in those studies had low support in many nodes. Utilization of 13 protein coding genes of the mitochondrial genome helped us to obtain a better supported tree of Baikalian amphipods in comparison with the ones described in previous molecular studies. The relatively small number of taxa sampled in our study does not allow us to rule out the possibility that the Baikalian clade might still be split into two or more independent lineages if more species are added to the analysis, particularly if *G. lacustris* is included. However, presently our data suggest that the common outgroup to all Baikalian species is the nearest sister species *G. duebeni*. This result corroborates the recent study by Hou and Sket (2016) [32].

The phylogenetic tree demonstrates separation of Baikalian amphipod species into two clades where the one includes species from the Micruropodidae and Crypturopodidae families and the other includes species from the Acanthogammaridae, Pallaseidae, and Eulimnogammaridae families (Fig. 3). This clusterization is in concordance with the current view on the existence of two major amphipod lineages in Lake Baikal, which is based on morphological investigations [25] and molecular phylogenetic data [30–32, 35].

The phylogenetic tree obtained in our study can be used to make some assumptions about the origination of certain ecological features of Baikalian amphipods. The Baikalian amphipods *C. tuberculatus*, *L. vortex* and *G. fasciatus*, which comprise one of the lineages in our study (Fig. 3), are mainly shallow-water inhabitants, having smooth body and display tolerance to warm water [13, 14, 115, 116]. Ancestors of these species may have originated during the Tertiary period when the climate was warm [117] and therefore they may be warm-water relicts [14, 25]. The second lineage of Baikalian amphipods contains species that possess more diverse morphological features, lifestyle and ecological niches. The radiation of this lineage presumably coincided with the completion of climate cooling period in the late Eocene – early Oligocene according to Kamaltynov [14]. This lineage is subdivided into two clades: the first one includes armored and relatively shallow water species *P. kesslerii* (Pallaseidae) and *A. victorii* (Acanthogammaridae) [13, 14], the second clade consists of two armored deep-water predator species (*G. cabanisii* and *B. grewingkii)* and a derived clade of smooth littoral amphipods of Gen. *Eulimnogammarus* Bazikalova, 1945, which differ from the rest by their feeding habits. The separation of species from the second lineage into two clades is also supported by morphological features, as *A.victorii* and *P.kesslerii* possess more plesiomorphic traits in comparison to the rest of the species [13, 14]. The topology of the BI tree shows that the species of *Eulimnogammarus* genus may have originated from an ancestor inhabiting the abyssal zone of Lake Baikal. However, it is possible that an expanded sampling of Baikalian species may yet show the different trends of origination of the extant amphipods inhabiting different depths of the lake.

The topology of the BI tree revealed the paraphyly of family Acanthogammaridae, which in our study includes *A.victorii, B.grewingkii* and *G.cabanisii.* The phylogenetic separation of species within the Acanthogammaridae family (according to the contemporary taxonomy) was also observed in previous studies, though the trees in those studies were poorly resolved [28, 30, 35]. The Acanthogammaridae includes species with diverse morphological and ecological features united on the basis of armament characteristics [14]. However, according to Kamaltynov, the taxonomy of contemporary Acanthogammaridae still needs refinement. Some genera have features that may be sufficient for establishing new families. Additional molecular phylogenetic data including some nuclear markers and larger sampling of species from the Acanthogammaridae is necessary for the description of new families.

## Conclusion

The detailed comparative study of mitochondrial genomes of endemic Baikalian amphipods belonging to the most diverse species flock of Lake Baikal is reported. The newly sequenced complete and nearly complete mitochondrial genomes of seven Baikalian species were presented in this study i.e. *Acanthogammarus victorii, Crypturopus tuberculatus, Eulimnogammarus cyaneus, Garjajewia cabanisii, Gmelinoides fasciatus, Linevichella vortex and Pallaseopsis kesslerii*.

The examined mitochondrial genome sequences were used to resolve phylogenetic relationships within the group of Baikalian amphipods. The branching order of the phylogenetic tree supports the separation of Baikalian species into two major lineages. Our data also support the paraphyly of Acanthogammaridae. The measurement of nucleotide diversity of protein-coding parts of Baikalian and non-Baikalian species allowed us to define the fragments of mitogenome most suitable for different scales of phylogenetic and/or population studies of amphipods.

The structural analysis of available Baikalian amphipod mitochondrial genomes revealed high variability in their length and gene order. Gene order of all mitochondrial genomes studied is changed relative to the pancrustacean ground pattern. Four species (*G.cabanisii, E.vittatus, A.victorii* and *G.fasciatus*) have extra tRNA genes copies. More severe rearrangements of protein coding genes and ribosomal RNA genes are found in *P. kesslerii, C. tuberculatus* and *G. fasciatus*. Multiple and unusually long intergenic spacers are found in mitochondrial genomes of *B.grewingkii, P.kesslerii* and *G.fasciatus*. Unusually high structural diversity of the amphipod mitochondrial genomes in Lake Baikal points at the possibility of high mutagenic pressure. This turns Baikalian amphipods into a potentially attractive model for studies of mitochondrial genome evolution in general.

## Methods

### Sampling, DNA extraction, mitochondrial genome amplification, sequencing

Amphipods species used for this study were collected in Lake Baikal from 2011 to 2013. Sampling was performed both manually at the water edge and by trawling from the ship at greater depths (Table 1). The species investigated in the current study were selected to maximally cover the range of species morphologies and ecological niches such as depth inhabited. Additional requirement was the ease of species diagnosis of the specimens.

Total DNA was extracted using modified CTAB method [118]. Depending on size of species we used either a whole animal or its part (a leg) for DNA extraction. The species identification was carried out using taxonomic system of Kamaltynov [17].

Total DNA samples of three out of seven amphipod species (*G. fasciatus, A. victorii* and *G. cabanisii*) was used directly for Illumina next generation sequencing libraries preparation. Paired-end libraries with insert size of 300 b.p. and 600 b.p. were prepared according to protocols provided by manufacturer for HiSeq (TruSeq DNA Sample Prep Kit) and MiSeq (Nextera DNA Library Preparation Kit) Systems (Illumina, San Diego, CA, USA).

Alternatively, DNA samples of other four species (*P. kesslerii, L. vortex, C. tuberculatus* and *E. cyaneus*) were used for long-range amplification of mitochondrial DNA. Mitochondrial genomes sequences were amplified as two fragments overlapping between *cox1* to *rrnL* genes (Additional file 9). Primers were designed based on the alignment of *cox1* and *rrnL* gene sequences of Baikalian amphipods available in GenBank. Additionally the universal Folmer’s primers were used [119]. The sequences of primers used in this study are available in Additional file 5.

Primers COI_L1 and 16S_H were used to amplify the c.a. 11Kb long (in all species) fragment spanning from *cox1* to *rrnL*. PCR was performed using EncycloPlus PCR kit (Eurogen, Moscow, Russia). Each reaction contained 1 μl of 10× Encyclo buffer, 0.2 μl of dNTP Mixture (10 mM each), 0.5 μl of each primer, 1 μl of template DNA (10–50 ng), 0.2 μl of Encyclo polymerase mix and 6.6 μl of sterile water up to 10 μl. Amplification was carried out under following conditions: 95 °C for 1 min., followed by 30 cycles of 93 °C for 15 s., 60 °C for 15 s., 72 °C for 9 min., with final elongation at 72 °C for 3 min. Different pairs of primers were used to amplify the second fragment spanning from *rrnL* to *cox1* in cases of different species as summarized in Additional file 5. PCR conditions were the same but the elongation times were decreased.

PCR products were purified by ethanol precipitation and were further utilized for libraries preparation using the Nextera DNA Library Preparation Kit (Illumina, San Diego, CA, USA) provided by manufacturer. The paired-end libraries with insert length of 600 b.p. were constructed and sequenced using MiSeq System (Illumina, San Diego, CA, USA).

### Reads processing, mitochondrial genomes assembly and annotation

All sets of reads were cleaned from adapters, and parts with a quality score below 15 were trimmed using Trimmomatic-0.32 [120]. De-novo assembly was carried out with SPAdes 3.0.0 assembler [121]. Scaffolds of mitochondrial genome in the assemblies were identified using BLAST [122] and the reference sequences of amphipod *E. verrucosus*. Complete and nearly complete amphipod mitochondrial genome sequences obtained after assemblies were further used as reference sequences for mapping reads of appropriate species using Bowtie2 2.1.0 [123]. All generated read alignment files were used for manual correction of errors in reference sequences and for estimation of coverage depth. Visualization of read alignment files was made by Tablet 1.13.07.31 [124].

An automatic annotation of mitochondrial genome sequences was performed using the MITOS pipeline with default settings [125]. GenomeView browser [126] was used for visualization of annotation files and manual correction of gene boundaries. PCGs boundaries were verified by comparison with the orthologs of other amphipods and also taking into account adjacent tRNA genes positions [61]. rRNA genes boundaries were identified by comparison to mitochondrial rRNA genes sequences of other amphipods. tRNA genes and their secondary structures were predicted by MiTfi [127] as part of the MITOS pipeline [125].

### Protein-coding genes analyses

Nucleotide composition and codon usage were calculated by The Sequence Manipulation Suite [128]. Effective number of codons was assessed using INCA 2.1 [129]. AT and GC skew of entire mitochondrial sequences were calculated using the following formulae: AT-skew = (A-T)/(A + T) and GC-skew = (G-C)/(G + C) [130]. Visualization of AT-skew and GC-skew plots as well as AT content plot for (+) strand were made by a custom Python script with a sliding window of 100 b.p. with steps of 10 b.p. Nucleotide diversity (Pi) was estimated for every protein-coding gene of Baikalian and non-Baikalian amphipods (see the list of species in Additional file 5) using DNAsp v.5 [131].

### Phylogenetic inference

Protein coding nucleotide sequences were used for the phylogenetic inferences. Non-Baikalian amphipods were represented in the dataset by a single sequence for each species available in GeneBank by March 2016 (Additional file 5).

Each protein coding gene sequence set was aligned separately in codon-based fashion with TranslatorX web server [132] using ClustalW algorithm and then the sets were concatenated using Seaview 4.5.4 [133]. A resultant alignment contained 11,013 characters.

The best model of nucleotide substitution (GTR + I + G) was chosen with jModelTest [134]. The phylogenetic trees were built by MrBayes v. 3.2.1. [135]. Four independent runs of four MCMC chains were performed. Chains were run for five million generations, the first 30% of generations were discarded as burn-in. The resultant phylogenetic tree was visualized in FigTree v.1.4.2. [136]. Metacrangonictidae clade was used as a root of the tree.

## Additional files

Additional file 1: AT content plots for the (+) strand of mitochondrial genomes of Baikalian amphipods under study. The beginning of every graph corresponds to the start of the *cox1* gene. Dashed lines indicate the places of disruptions in incompletely sequenced mitochondrial genomes. The putative CRs are marked by the colored boxes. (PDF 16,893 kb)
Additional file 2: AT-skew and GC-skew plots for the (+) strand of mitochondrial genomes of Baikalian amphipods under study. The beginning of every graph corresponds to the start of the *cox1* gene. Dashed lines indicate the places of disruptions in incompletely sequenced mitochondrial genomes. (PDF 18,605 kb)
Additional file 3: Organization of the mitochondrial genomes of Baikalian amphipods under study. The incomplete stop codons are labeled in the tables with parentheses. (XLSX 28 kb)
Additional file 4: Summary of codon usage in protein coding genes of mitochondrial genomes of studied Baikalian amphipods. Bold notations indicate species with incomplete protein coding sequences. Sign “#” indicates total number of certain codon in protein-coding sequences of every species; “%” indicates percent of certain codon in total coding sequence in every species. (XLSX 17 kb)
Additional file 5: Table S1. Primers used for amplification of Baikalian amphipod mitochondrial genomes. **Table S2.** Accession numbers of amphipod mitochondrial genomes from GenBank database used in the study (XLSX 11 kb)
Additional file 6: The predicted mitochondrial tRNAs secondary structures of Baikalian amphipods under study. (PDF 9263 kb)
Additional file 7: Nucleotide composition statistics for ribosomal RNA genes in mitochondrial genomes of studied Baikalian amphipods. Bold numbers indicate incomplete sequences. (XLSX 10 kb)
Additional file 8: Alignment of the putative CR sequence of *Garjajewia cabanisii* mitochondrial genome with its incompletely sequenced counterpart. Dashed lines indicate a missing sequence. (PNG 370 kb)
Additional file 9: The scheme of regions amplified by PCR in mitochondrial genomes of Baikalian amphipods. *Eulimnogammarus verrucosus* was used as an example of Baikalian amphipod. Protein-coding genes and ribosomal RNA genes are shown as sectors. Transfer RNA genes are labeled by their single-letter amino acid code. The features located on the (−) strand are shown inside the circle. The semicircles outside of the gene map denote the regions of the long-range amplification. (PNG 1167 kb)

## Abbreviations

BI: Bayesian inference
CR: Control region
ENC: Effective number of codons
PCGs: Protein-coding genes
