# Supplementary material for: Evolution of mitochondrial genomes in Baikalian amphipods
Source: BMC Genomics. 2016 Dec 28;17(Suppl 14):1016. doi: 10.1186/s12864-016-3357-z (PMC5249044; doi:10.1186/s12864-016-3357-z)

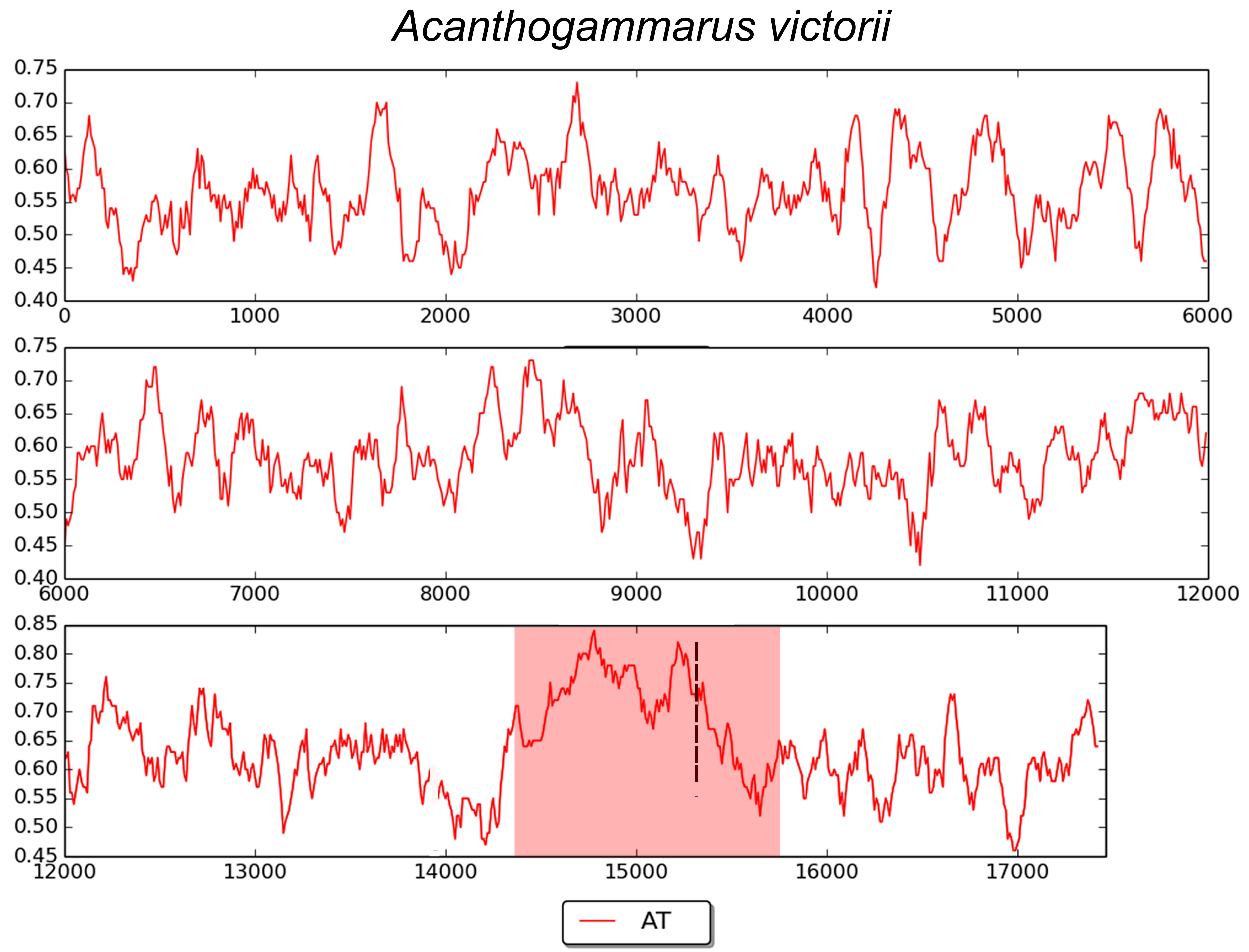
Acanthogammarus victorii
AT

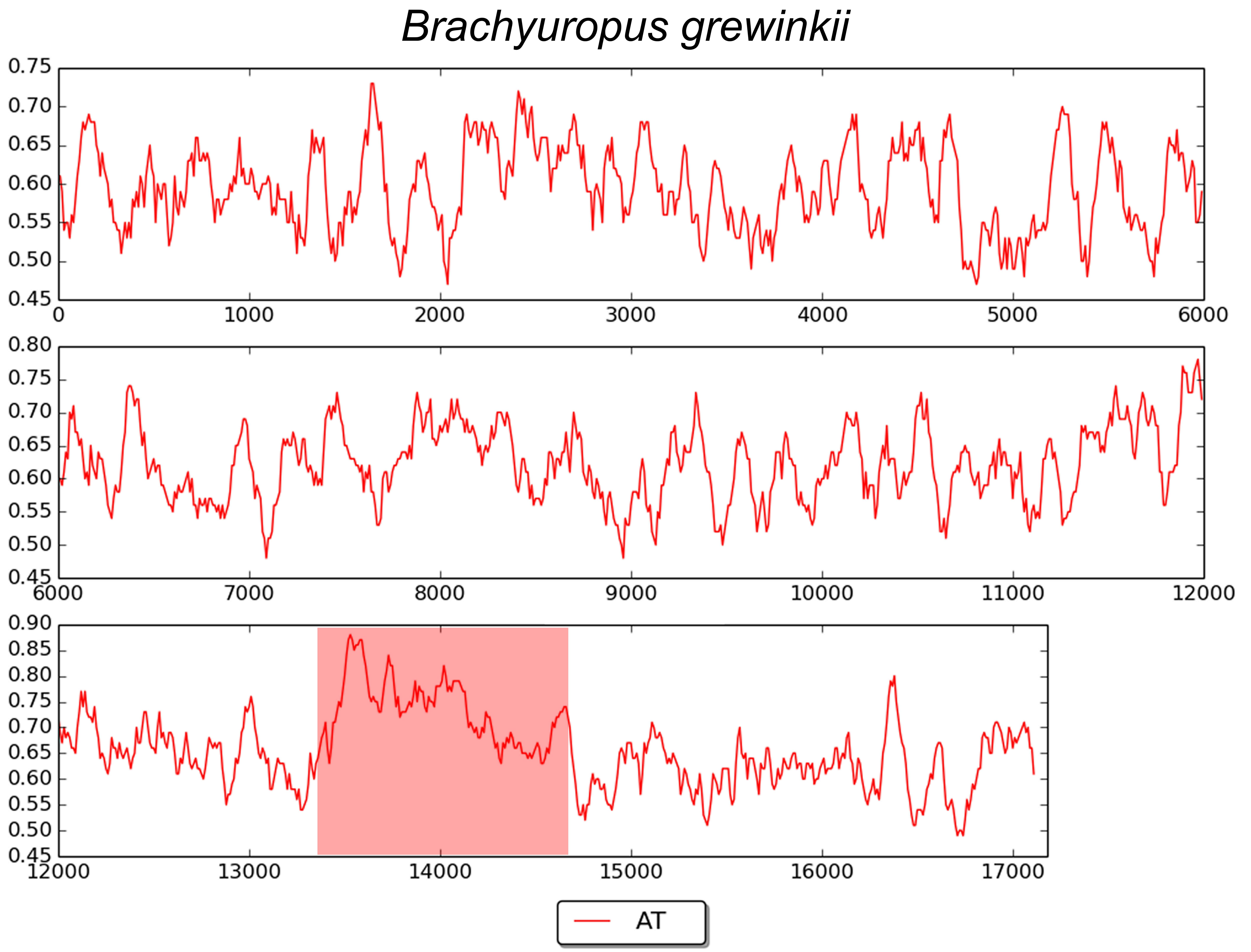
Brachyuropus grewinkii
AT

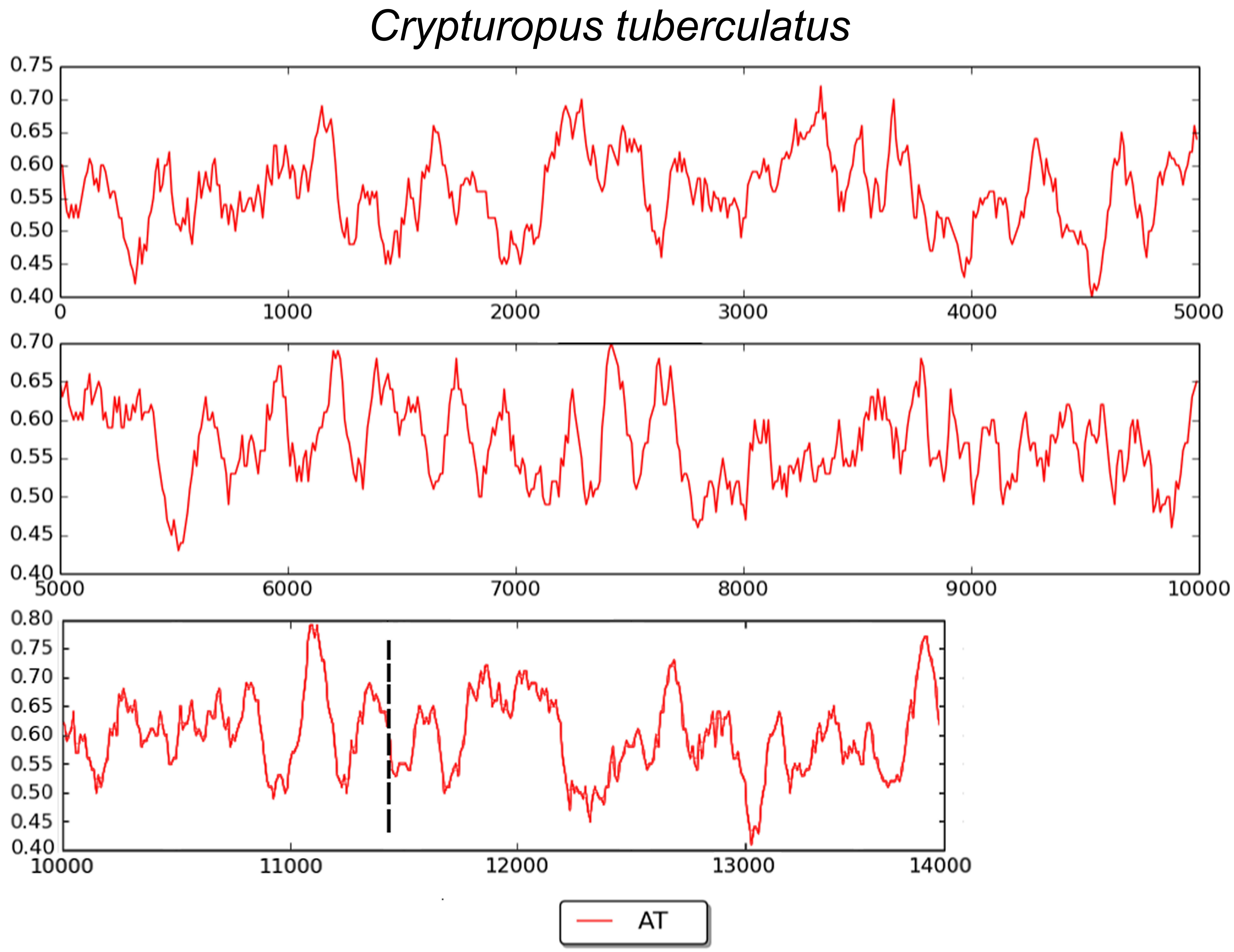
Crypturopus tuberculatus
0.75
0.70
0.65
0.60
0.55
0.50
0.45
0.40
0
1000
2000
3000
4000
5000
6000
7000
8000
9000
10000
0.80
11000
12000
13000
14000
AT

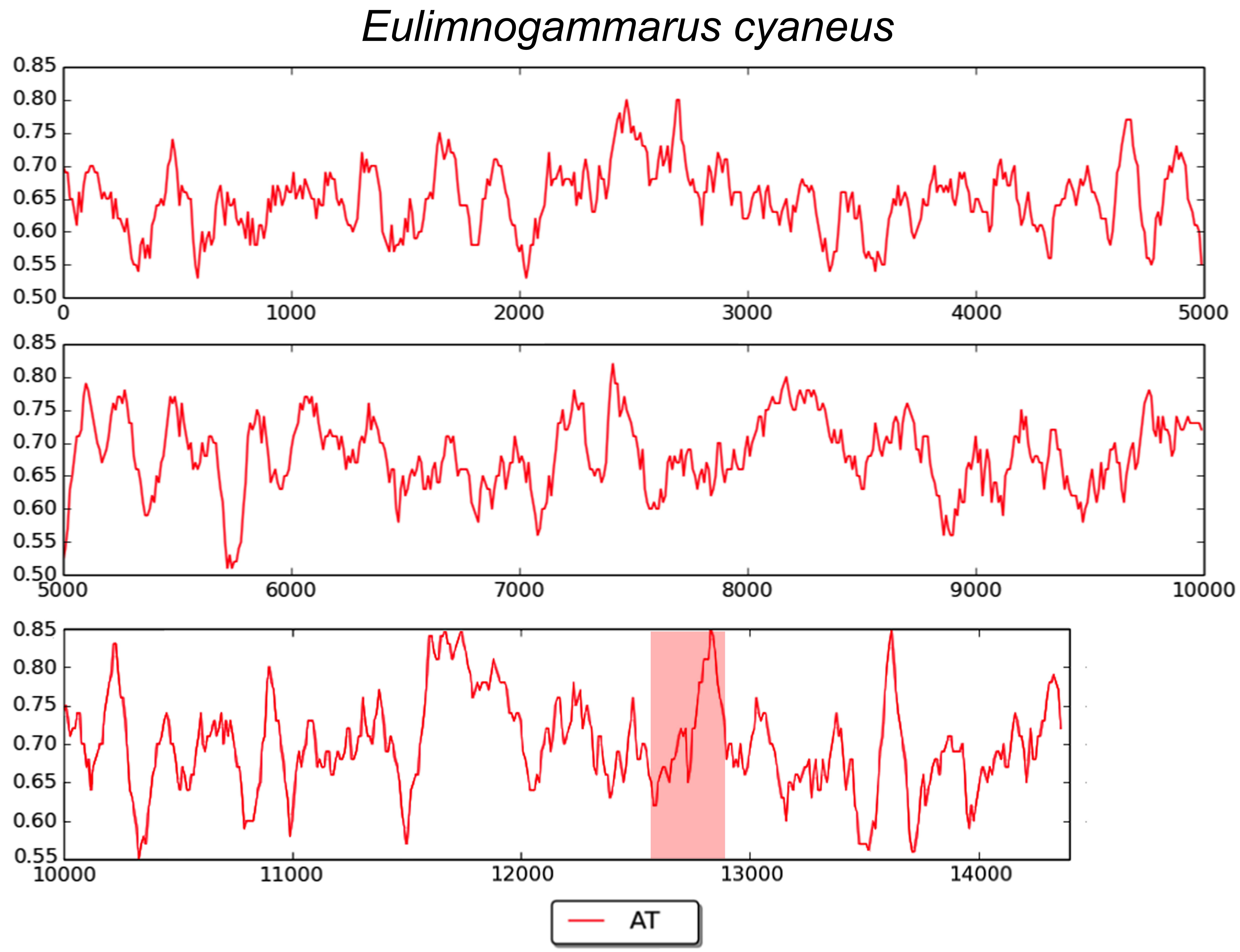
Eulimnogammarus cyaneus
0.85
0.80
0.75
0.70
0.65
0.60
0.55
0.50
0
1000
2000
3000
4000
5000
6000
7000
8000
9000
10000
11000
12000
13000
14000
AT

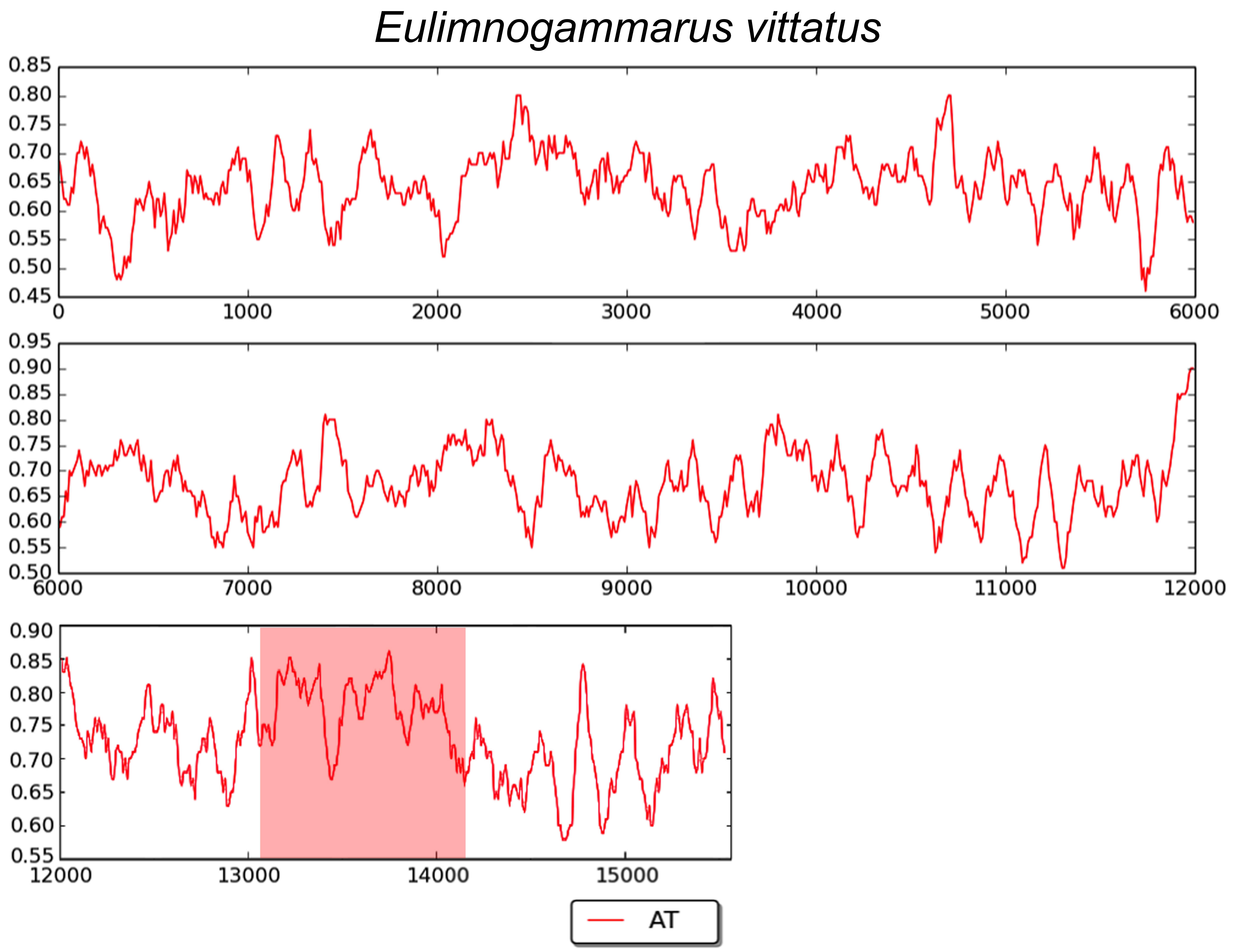
Eulimnogammarus vittatus
AT

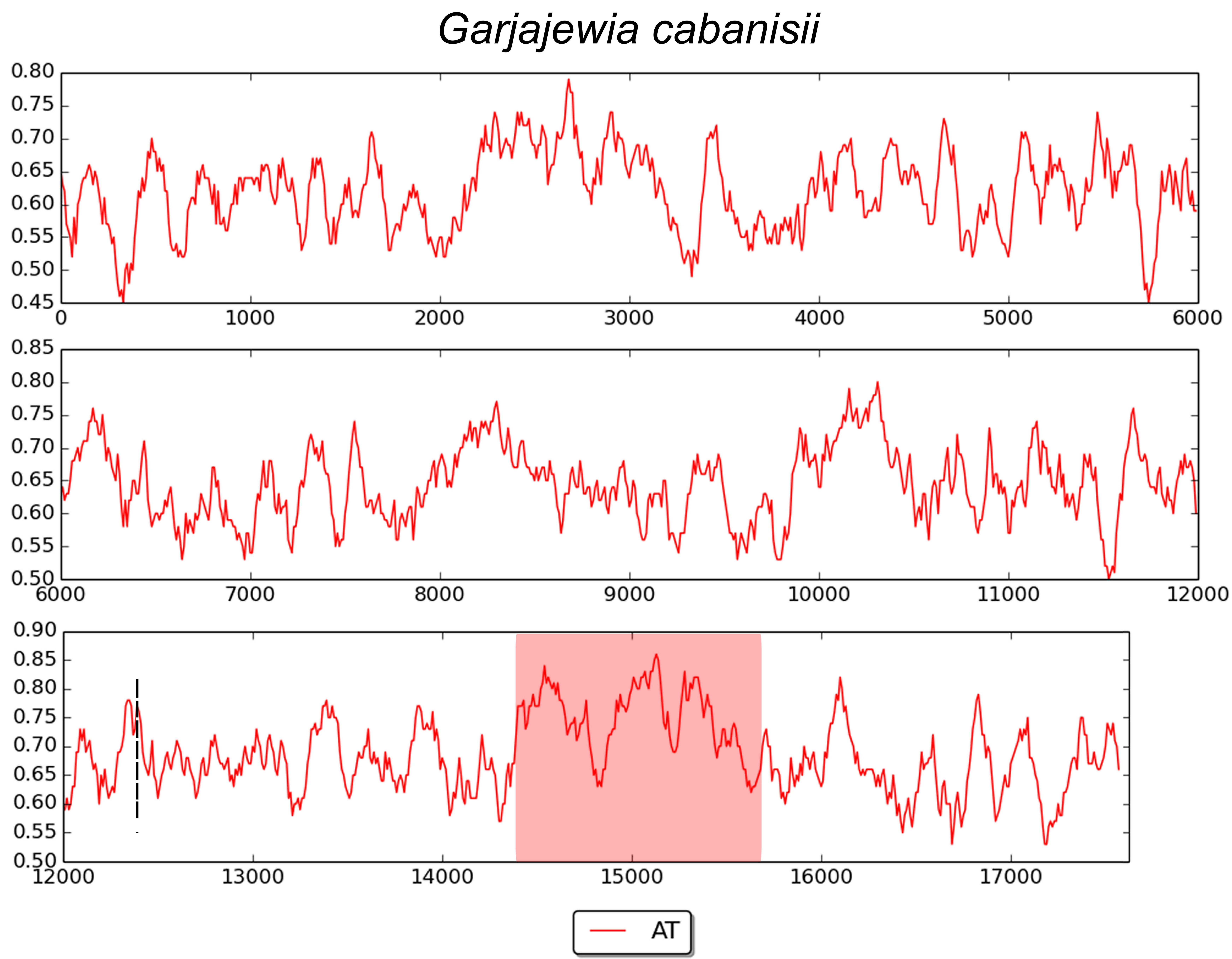
Garjajewia cabanisii
AT

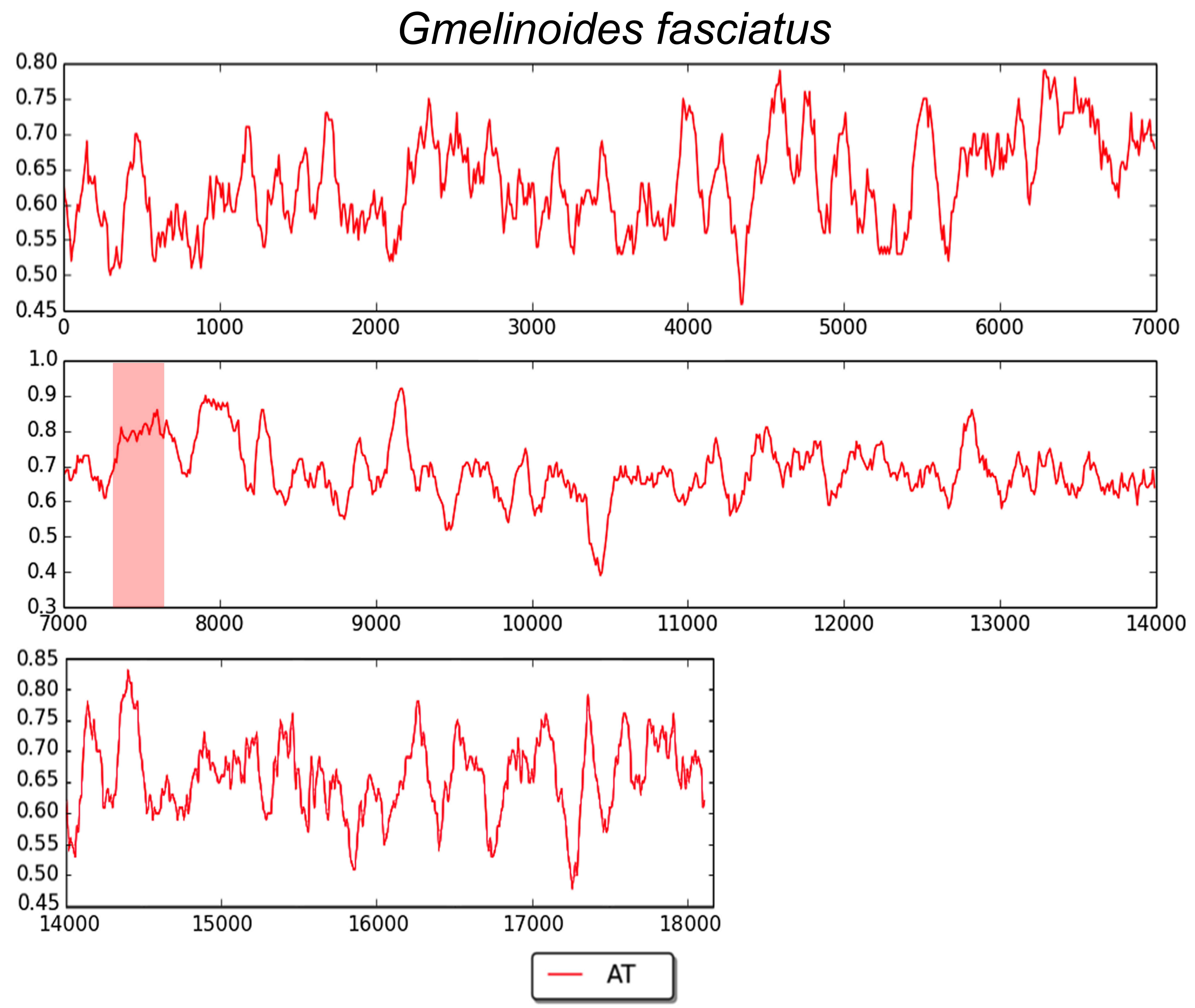
Gmelinoides fasciatus
AT

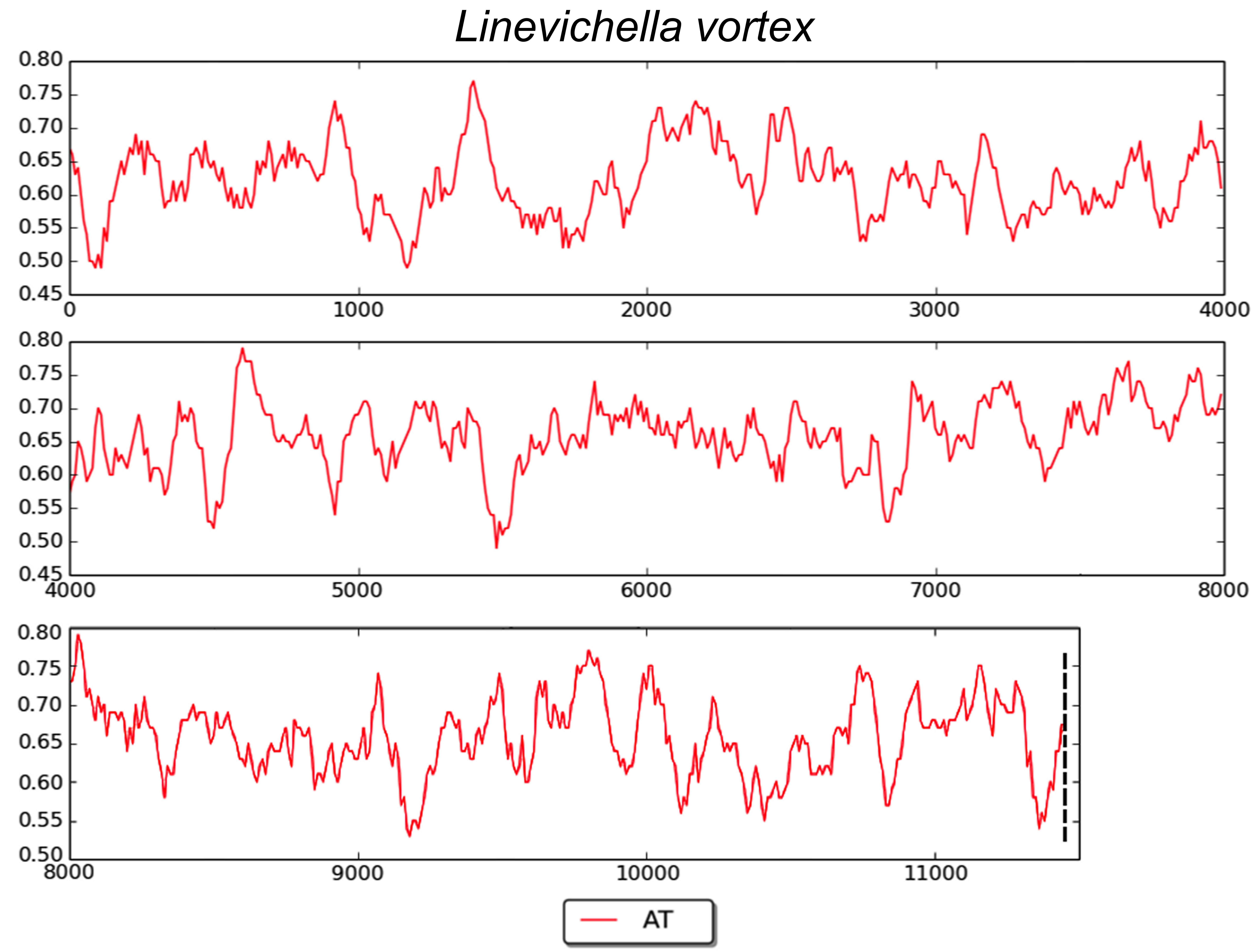
Linevichella vortex
AT

Pallaseopsis kesslerii

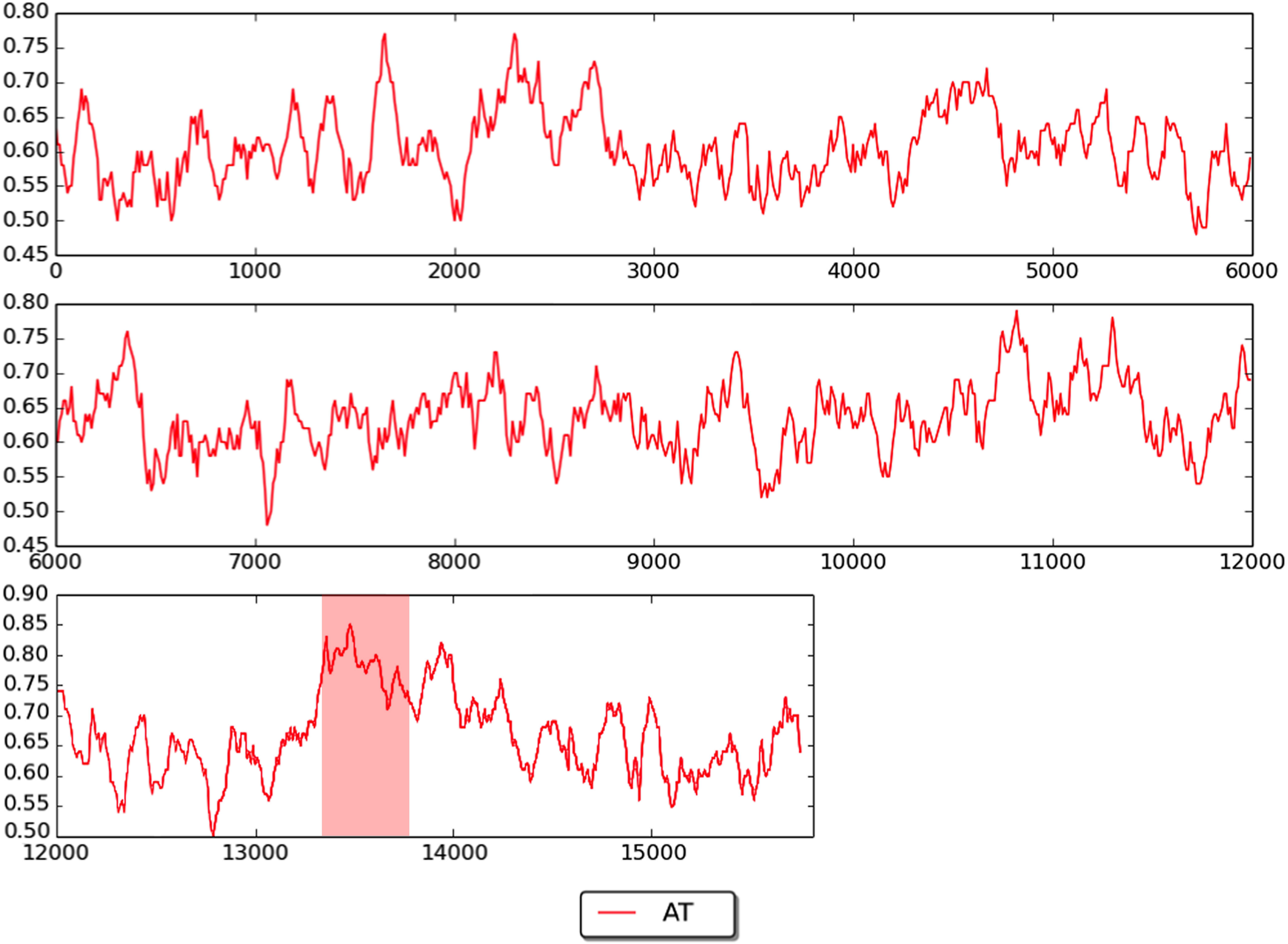

Supplement: Additional file 1: — AT content plots for the (+) strand of mitochondrial genomes of Baikalian amphipods under study. The beginning of every graph corresponds to the start of the cox1 gene. Dashed lines indicate the places of disruptions in incompletely sequenced mitochondrial genomes. The putative CRs are marked by the colored boxes. (PDF 16,893 kb) [file 12864_2016_3357_MOESM1_ESM.pdf]
