# Supplementary material for: Evolution of mitochondrial genomes in Baikalian amphipods
Source: BMC Genomics. 2016 Dec 28;17(Suppl 14):1016. doi: 10.1186/s12864-016-3357-z (PMC5249044; doi:10.1186/s12864-016-3357-z)
Supplement: Additional file 2: — AT-skew and GC-skew plots for the (+) strand of mitochondrial genomes of Baikalian amphipods under study. The beginning of every graph corresponds to the start of the cox1 gene. Dashed lines indicate the places of disruptions in incompletely sequenced mitochondrial genomes. (PDF 18,605 kb) [file 12864_2016_3357_MOESM2_ESM.pdf]

## *Acanthogammarus victorii*

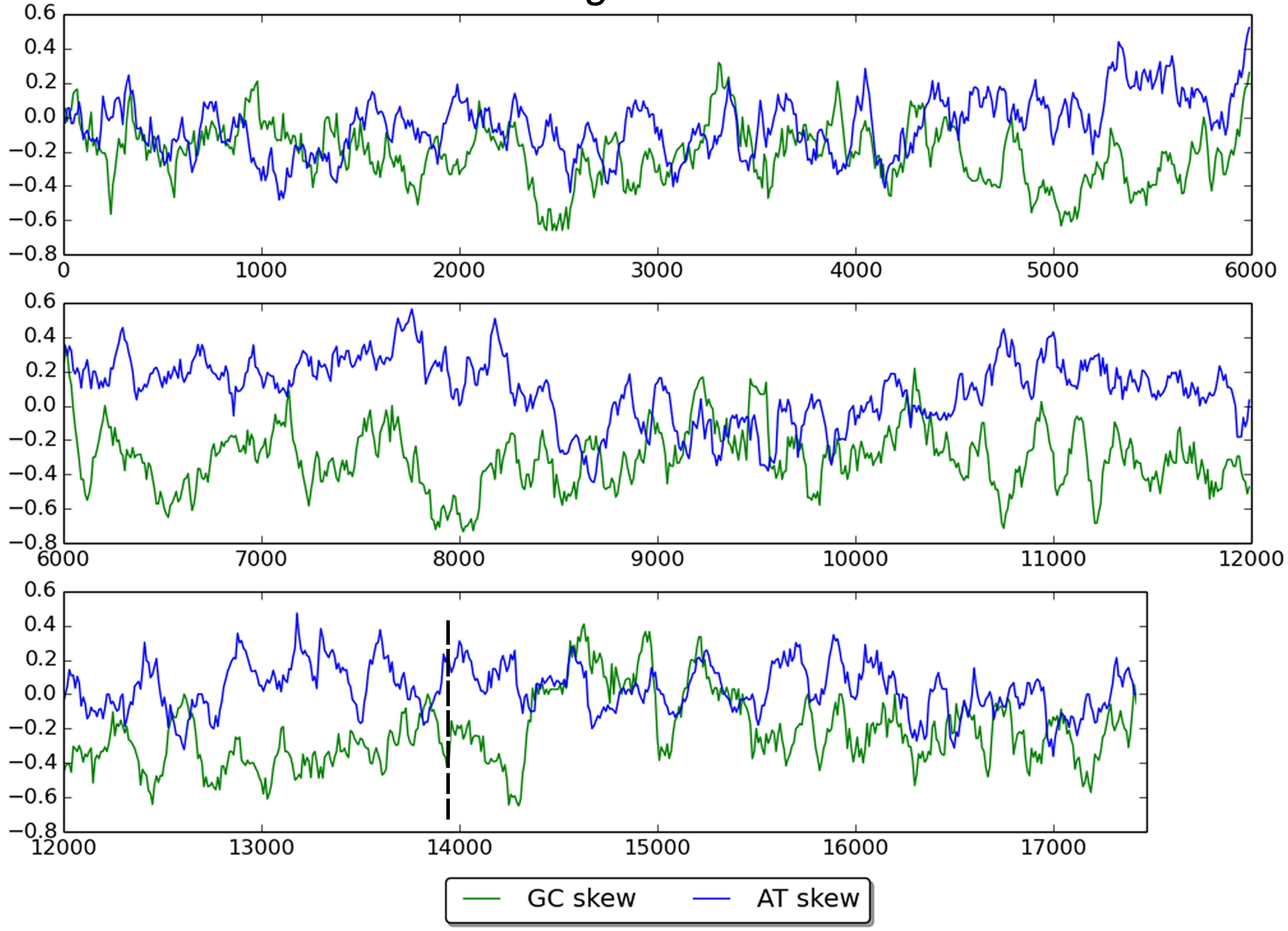


## *Brachyuropus grewingkii*

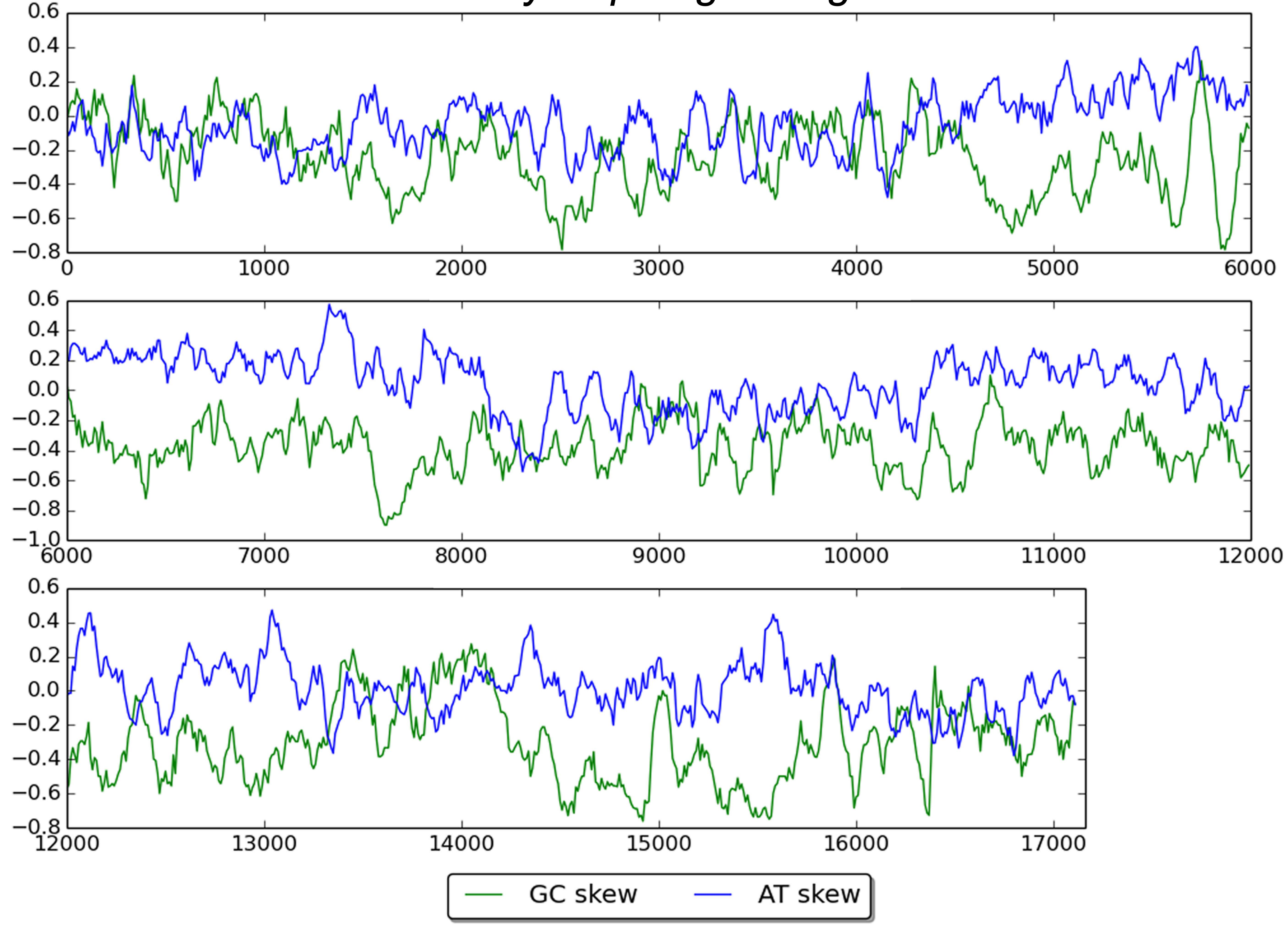

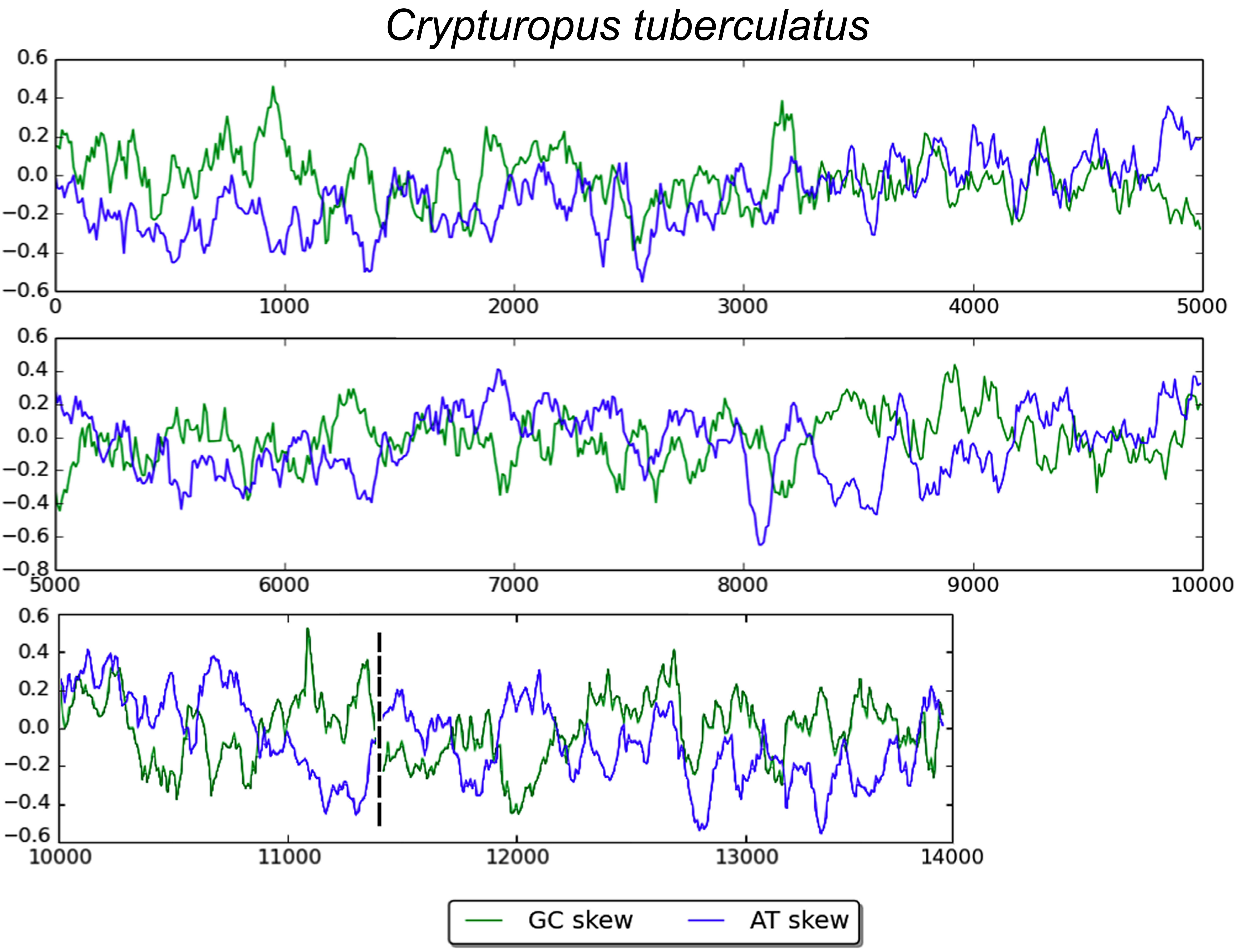
Crypturopus tuberculatus
GC skew
AT skew

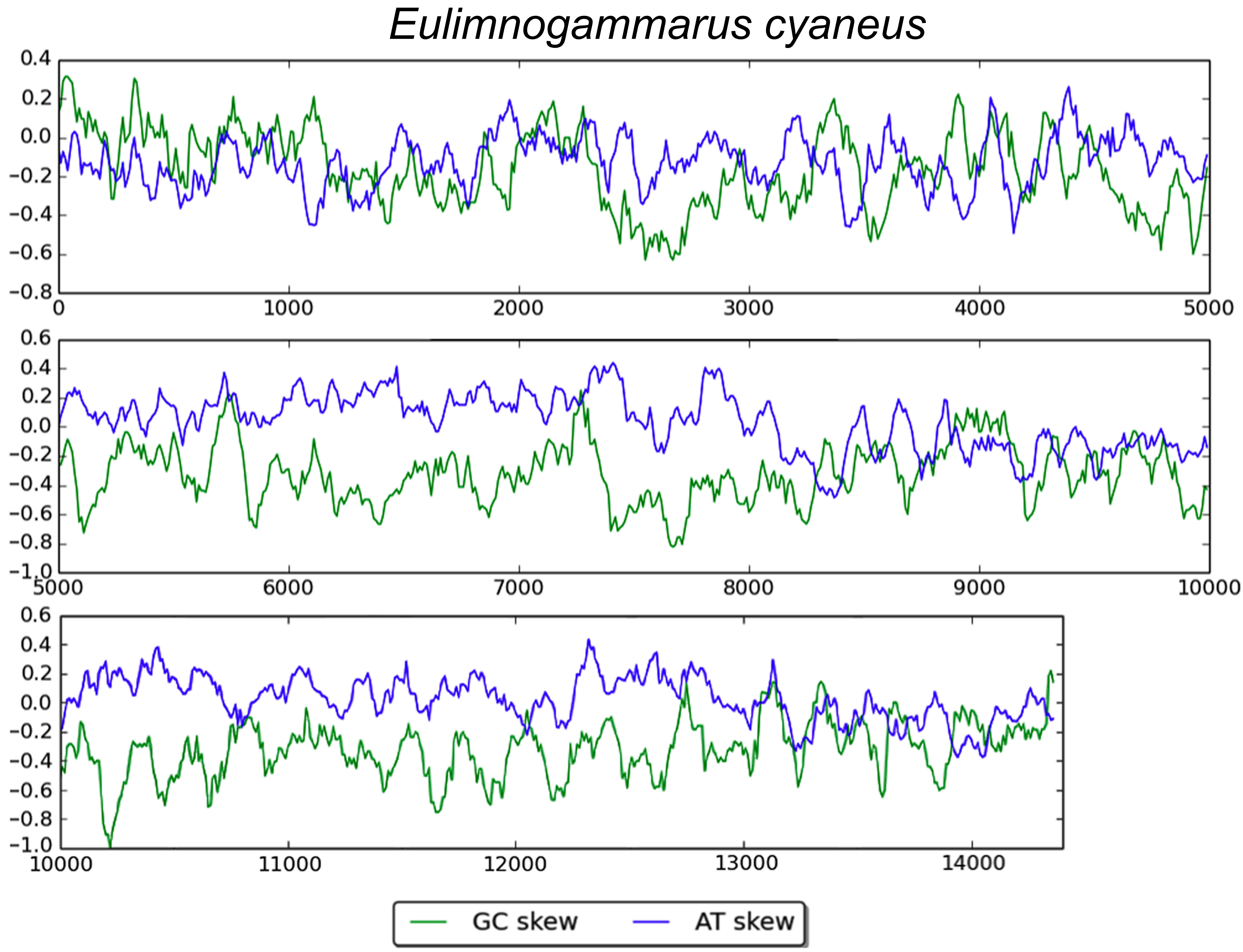
Eulimnogammarus cyaneus
GC skew
AT skew

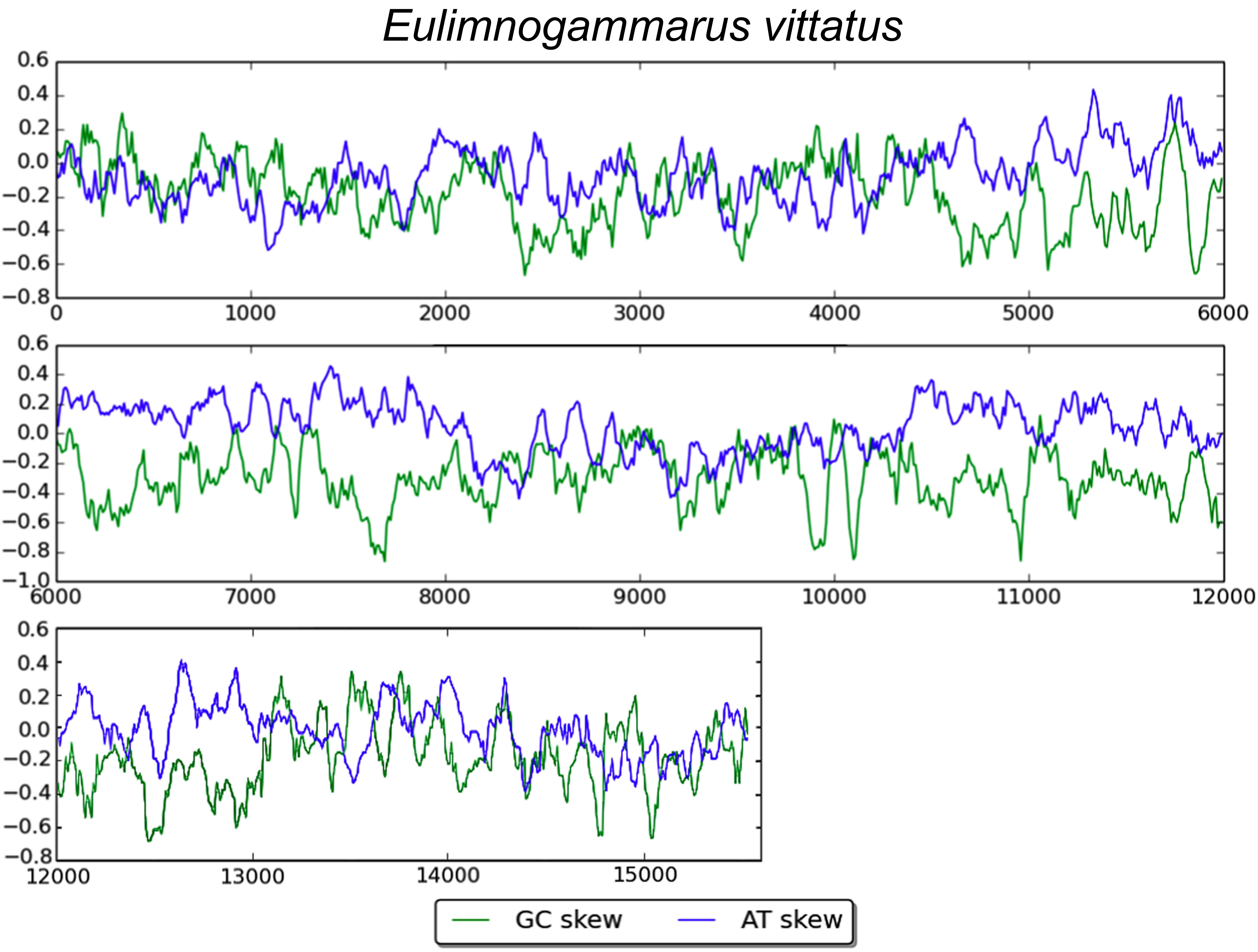
Eulimnogammarus vittatus
GC skew
AT skew

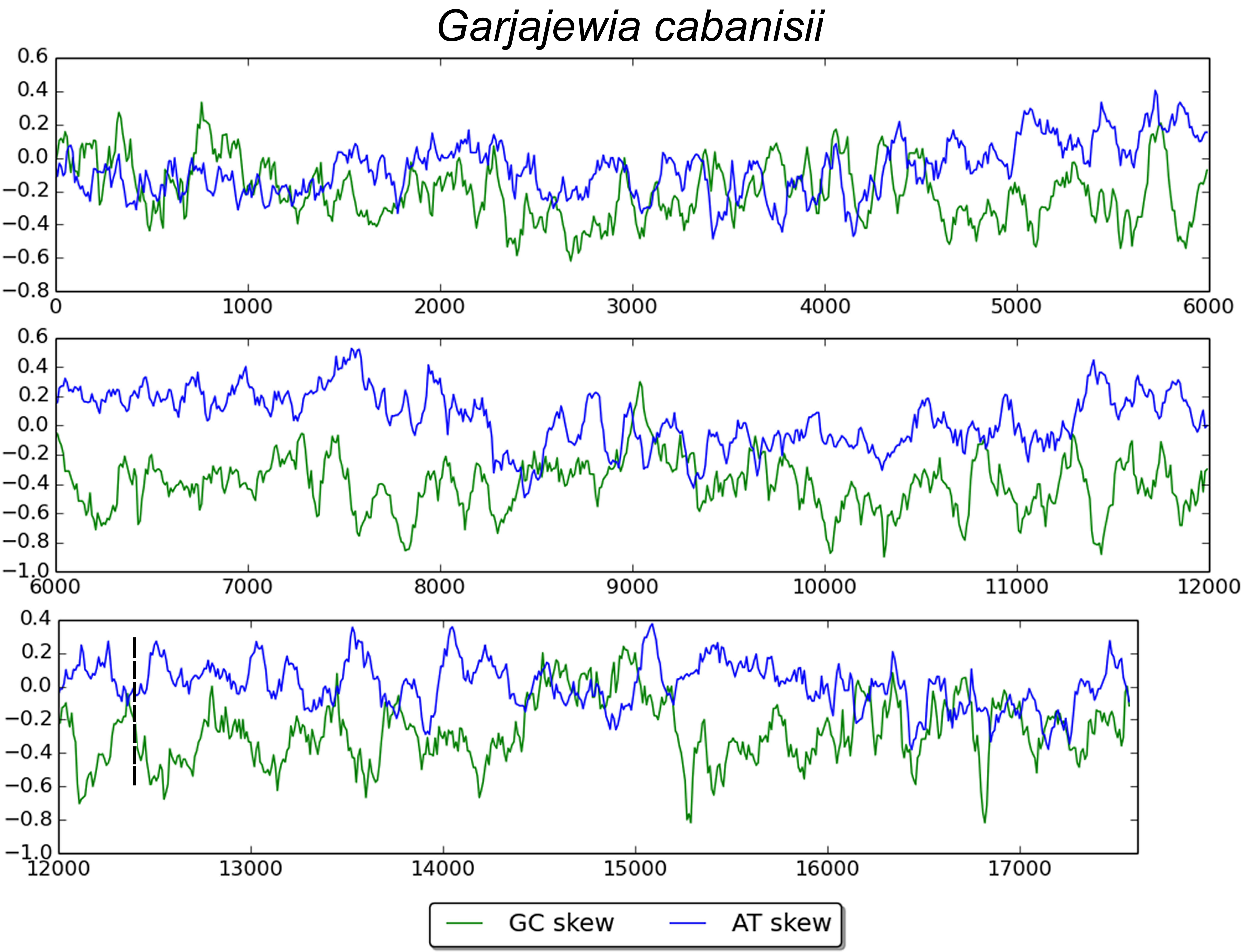
Garjajewia cabanisii
GC skew
AT skew

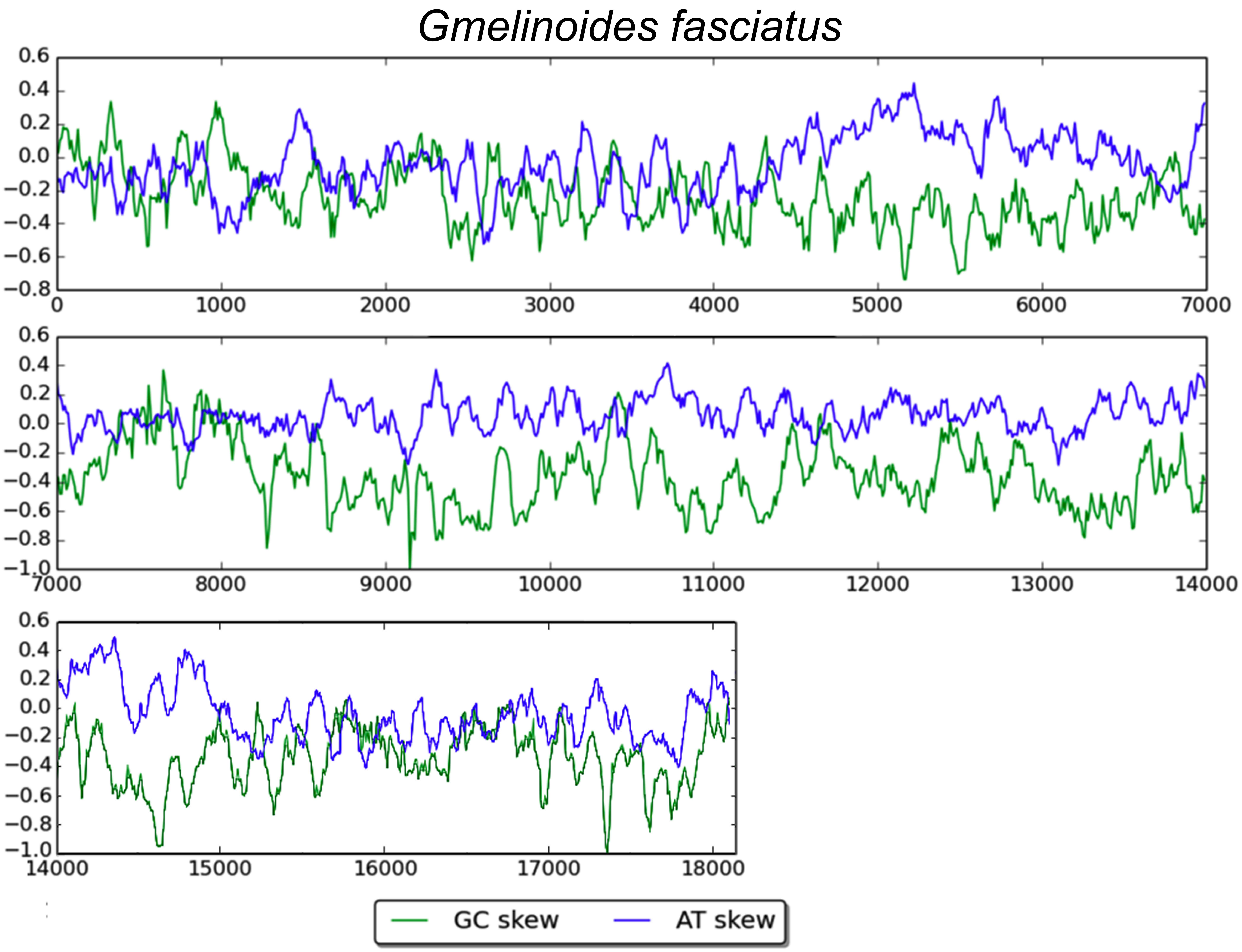
Gmelinoides fasciatus
GC skew
AT skew

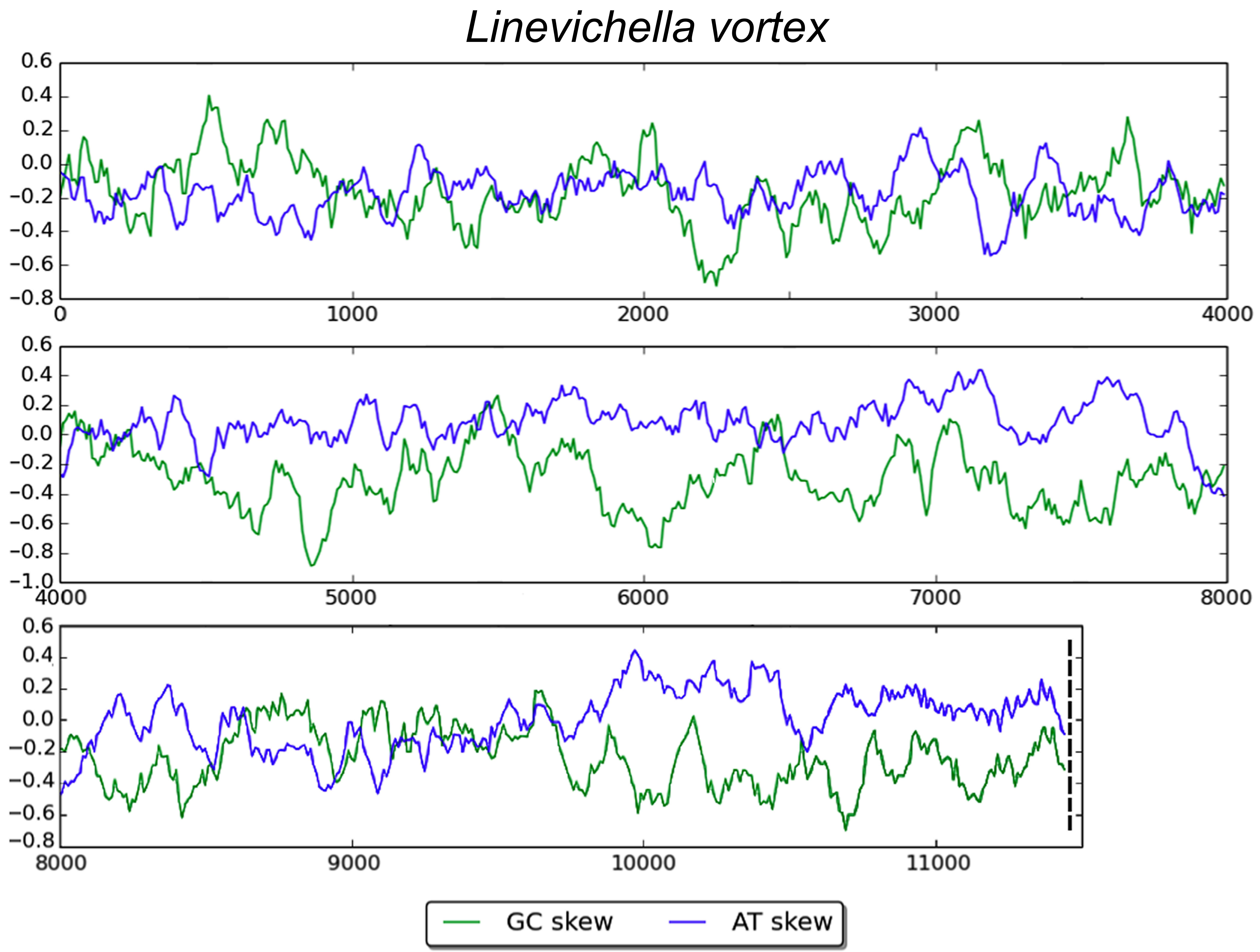
Linevichella vortex
GC skew
AT skew

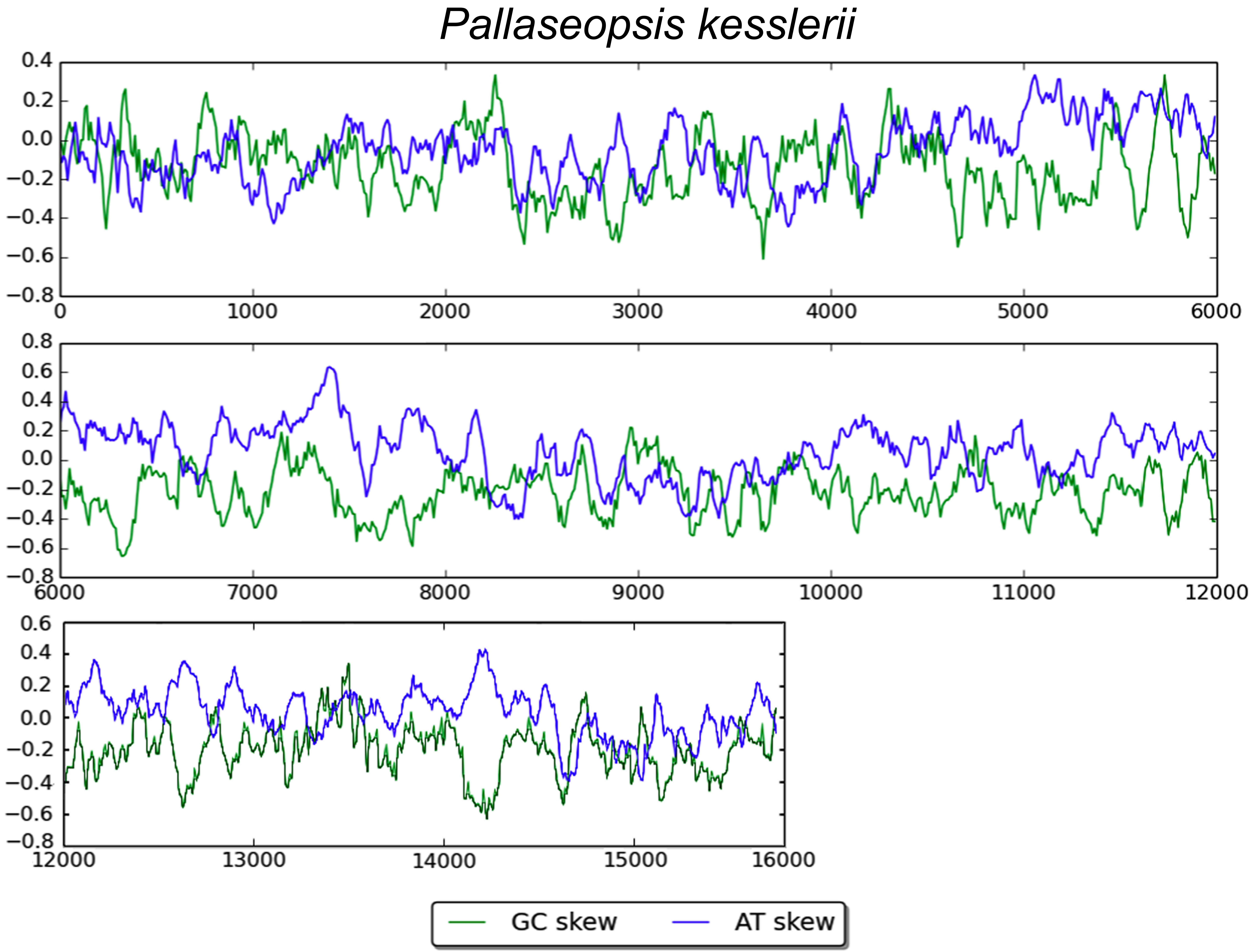
Pallaseopsis kesslerii
GC skew
AT skew
