# Supplementary material for: Evolution of mitochondrial genomes in Baikalian amphipods
Source: BMC Genomics. 2016 Dec 28;17(Suppl 14):1016. doi: 10.1186/s12864-016-3357-z (PMC5249044; doi:10.1186/s12864-016-3357-z)

Acanthogammarus victorii

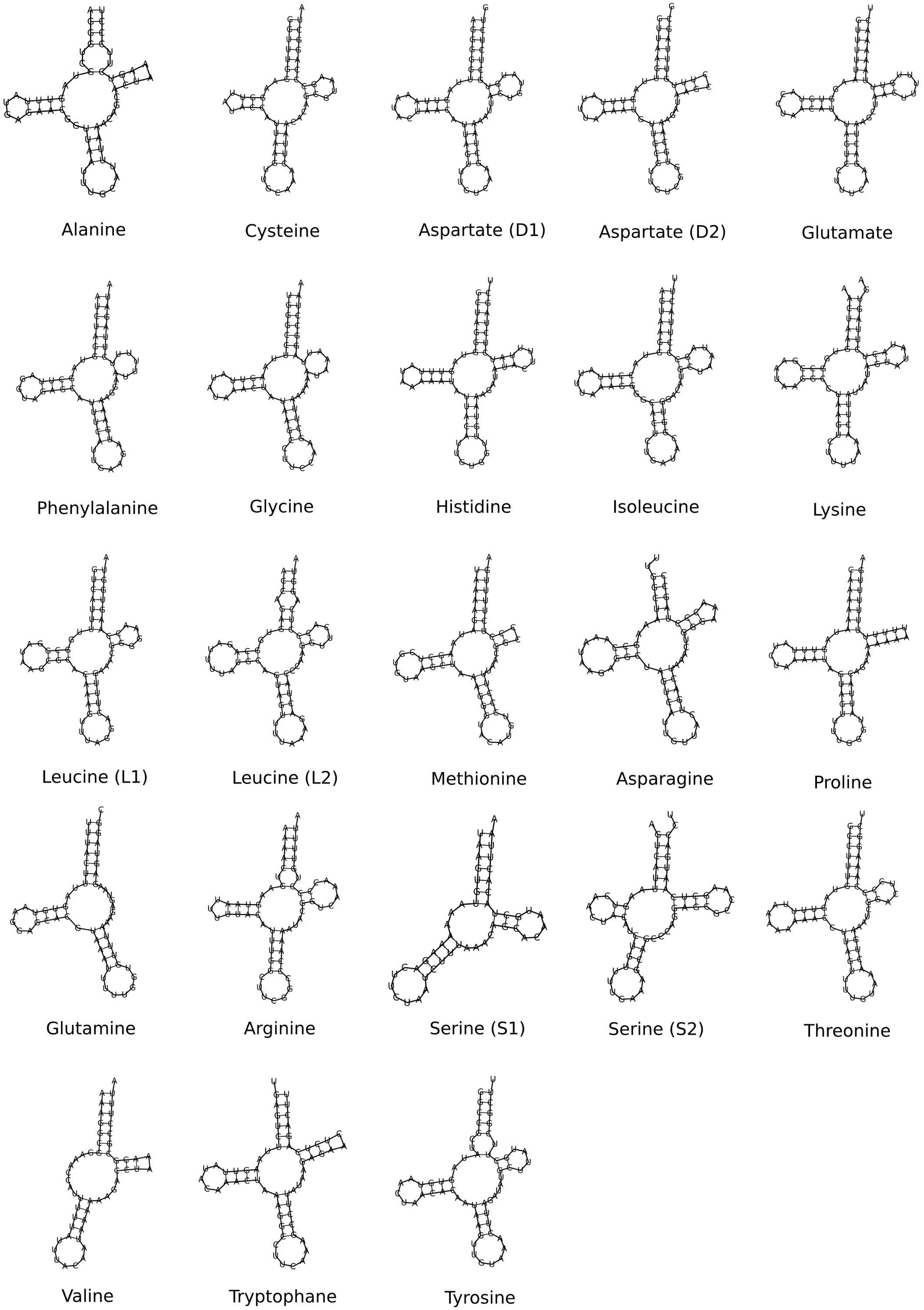

Brachyuropus grewinkii

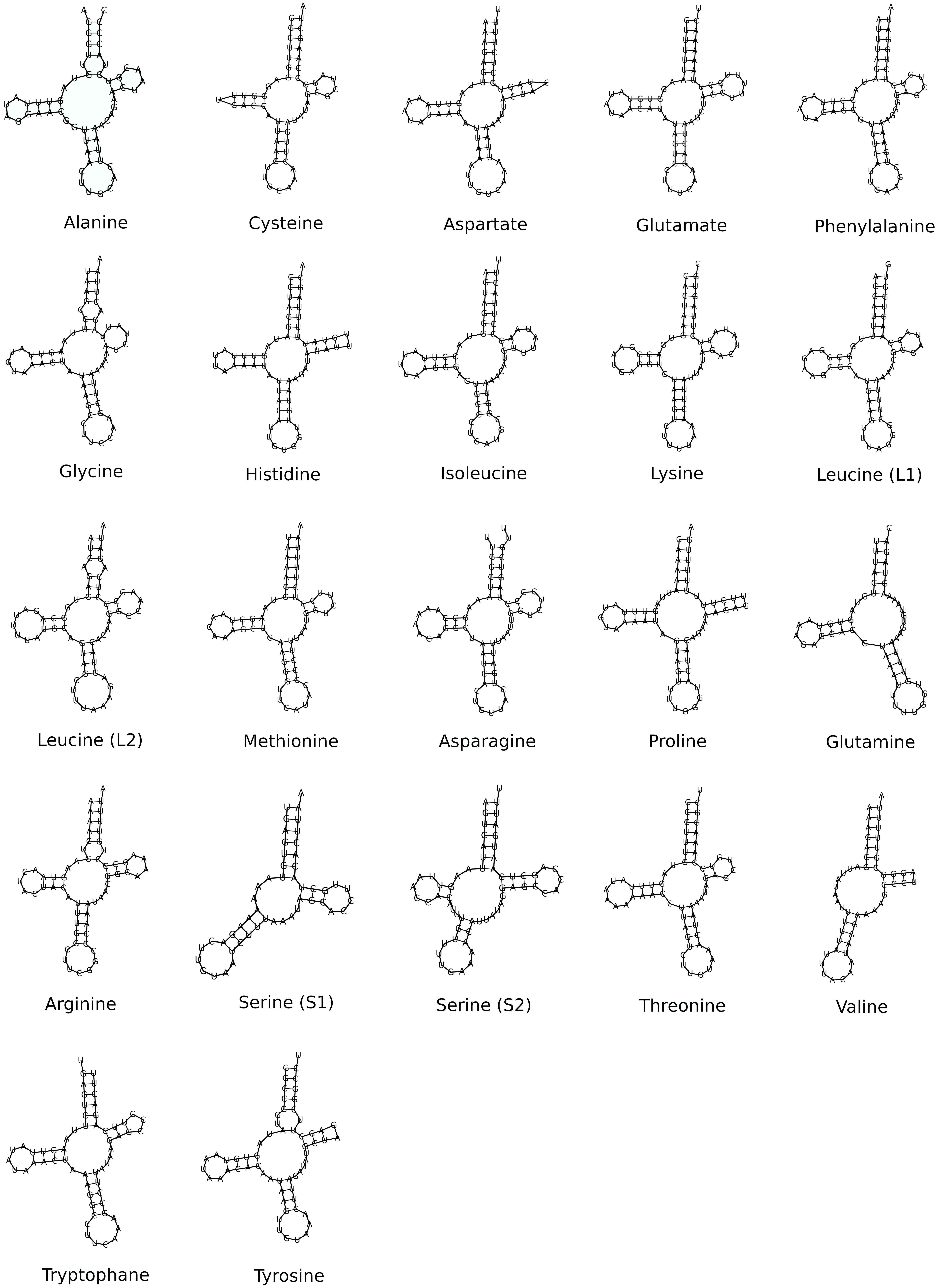

Crypturopus tuberculatus

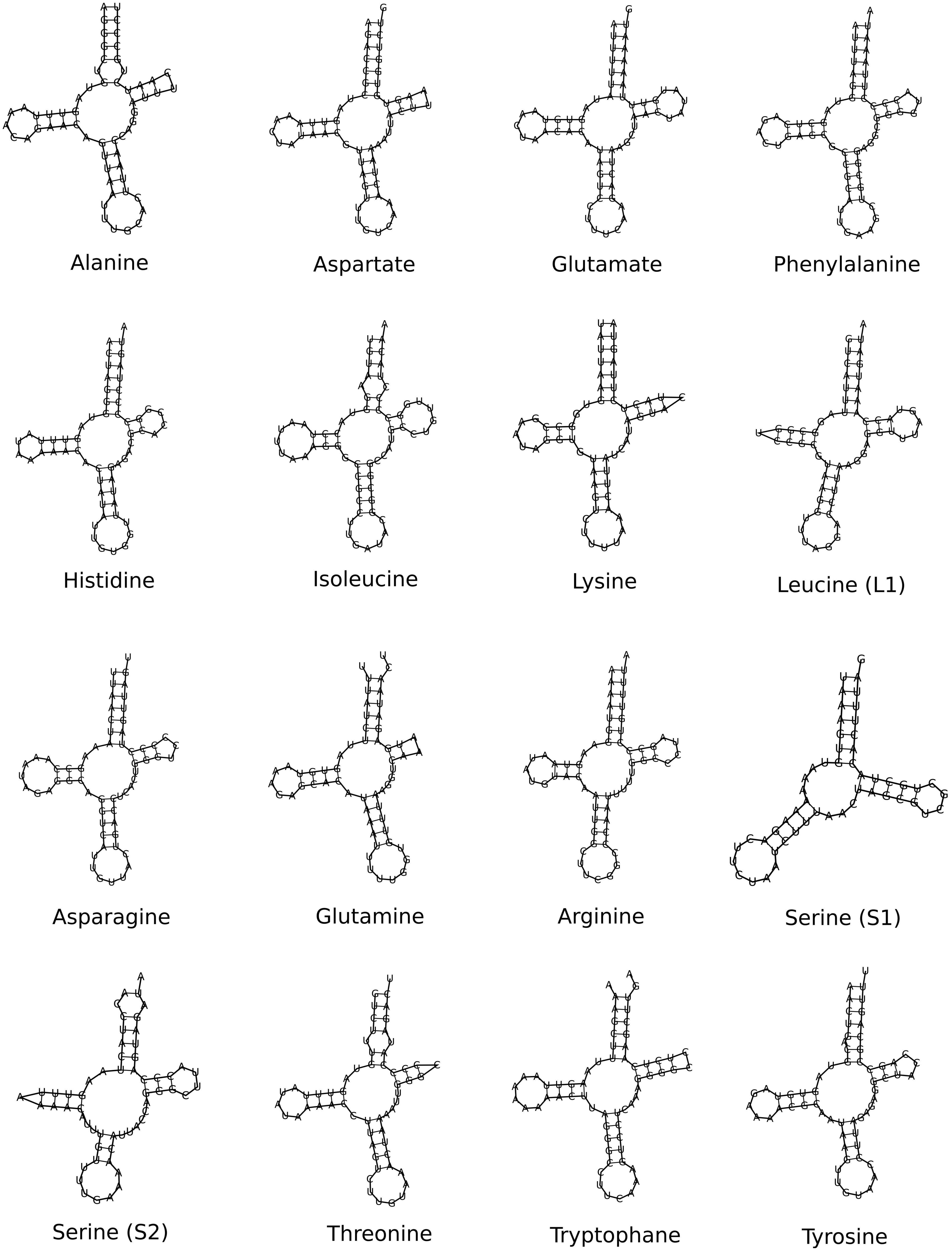

Eulimnogammarus cyaneus

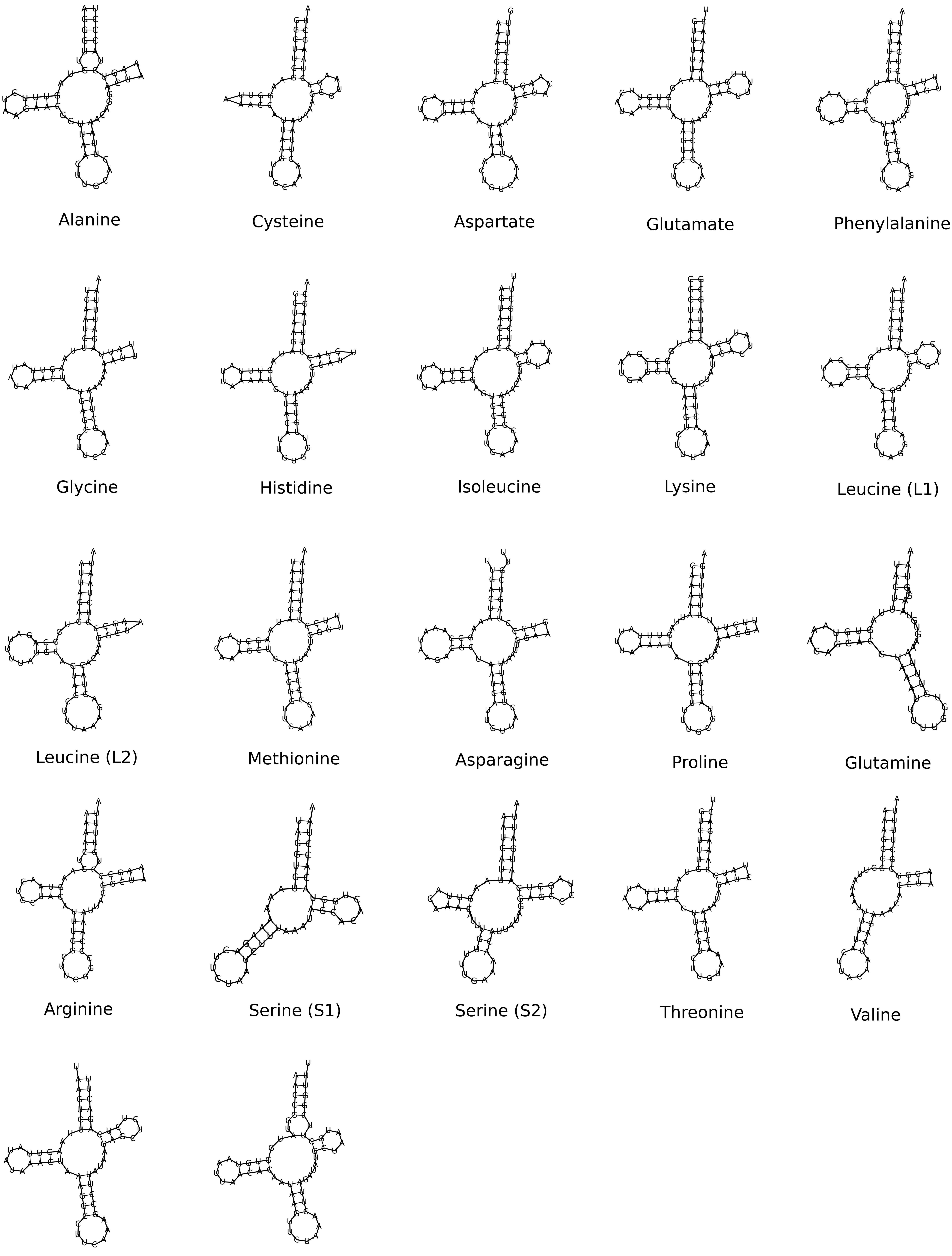

Eulimnogammarus vittatus

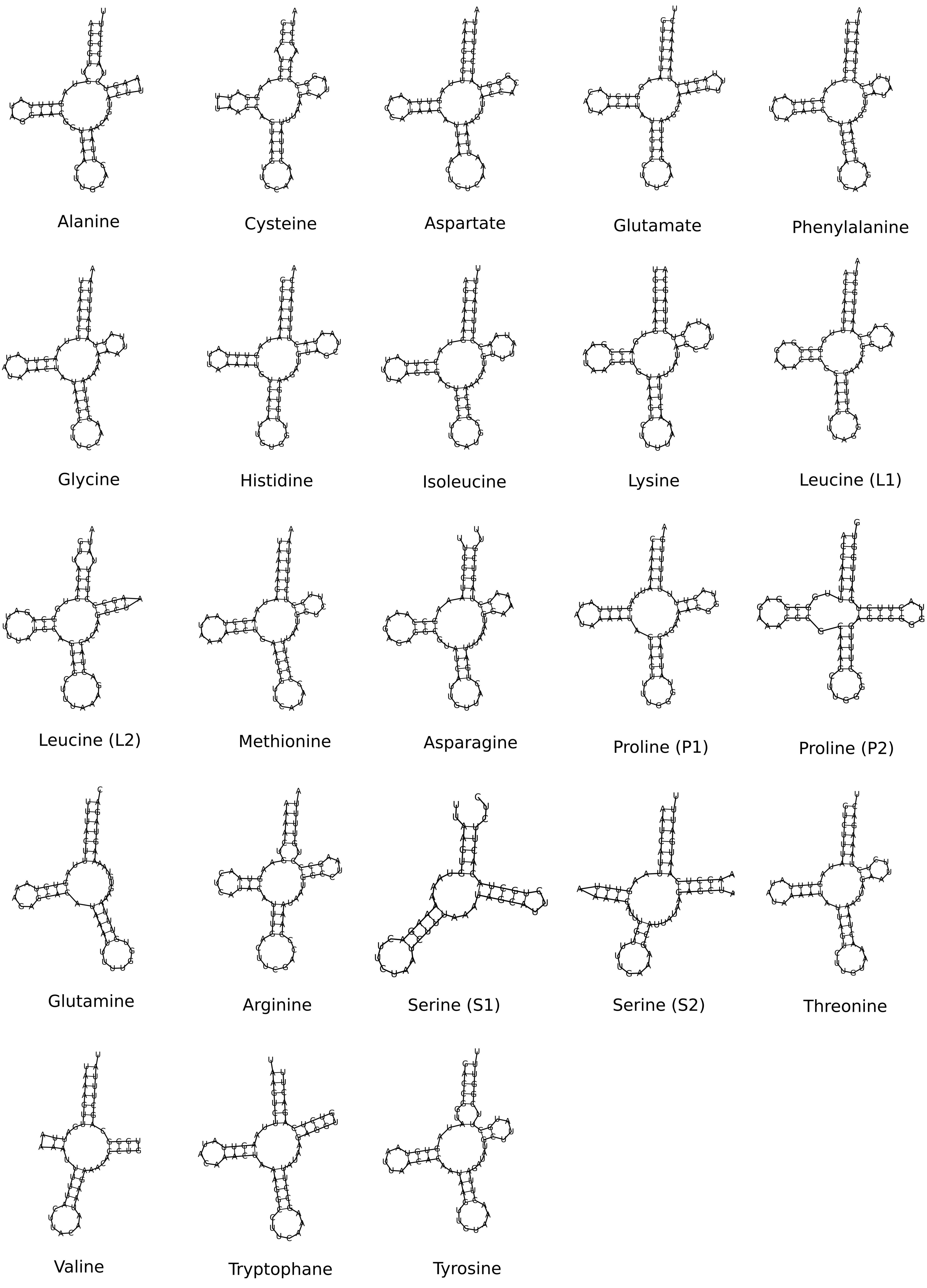

Garjajewia cabanisii

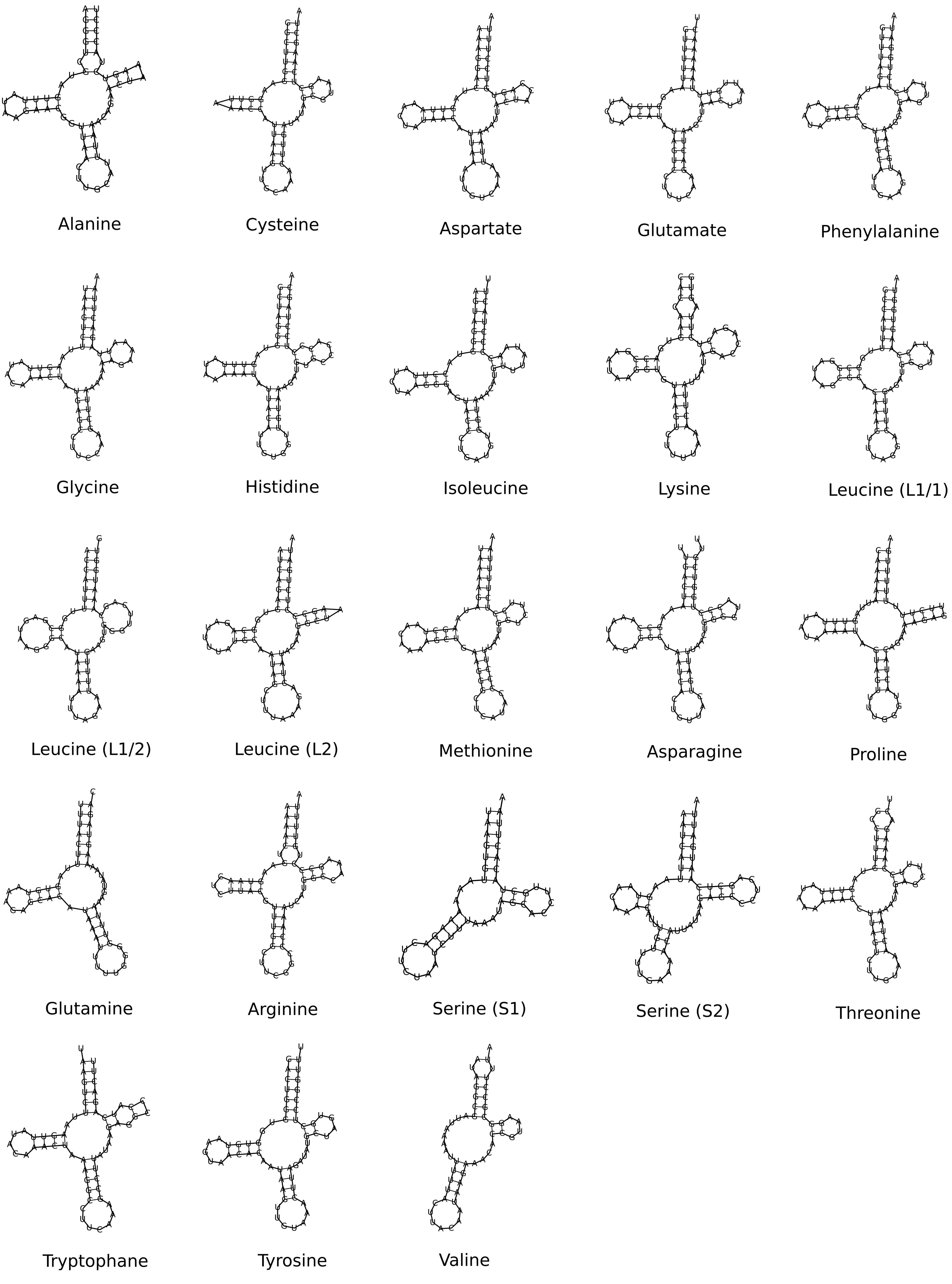

Gmelinoides fasciatus

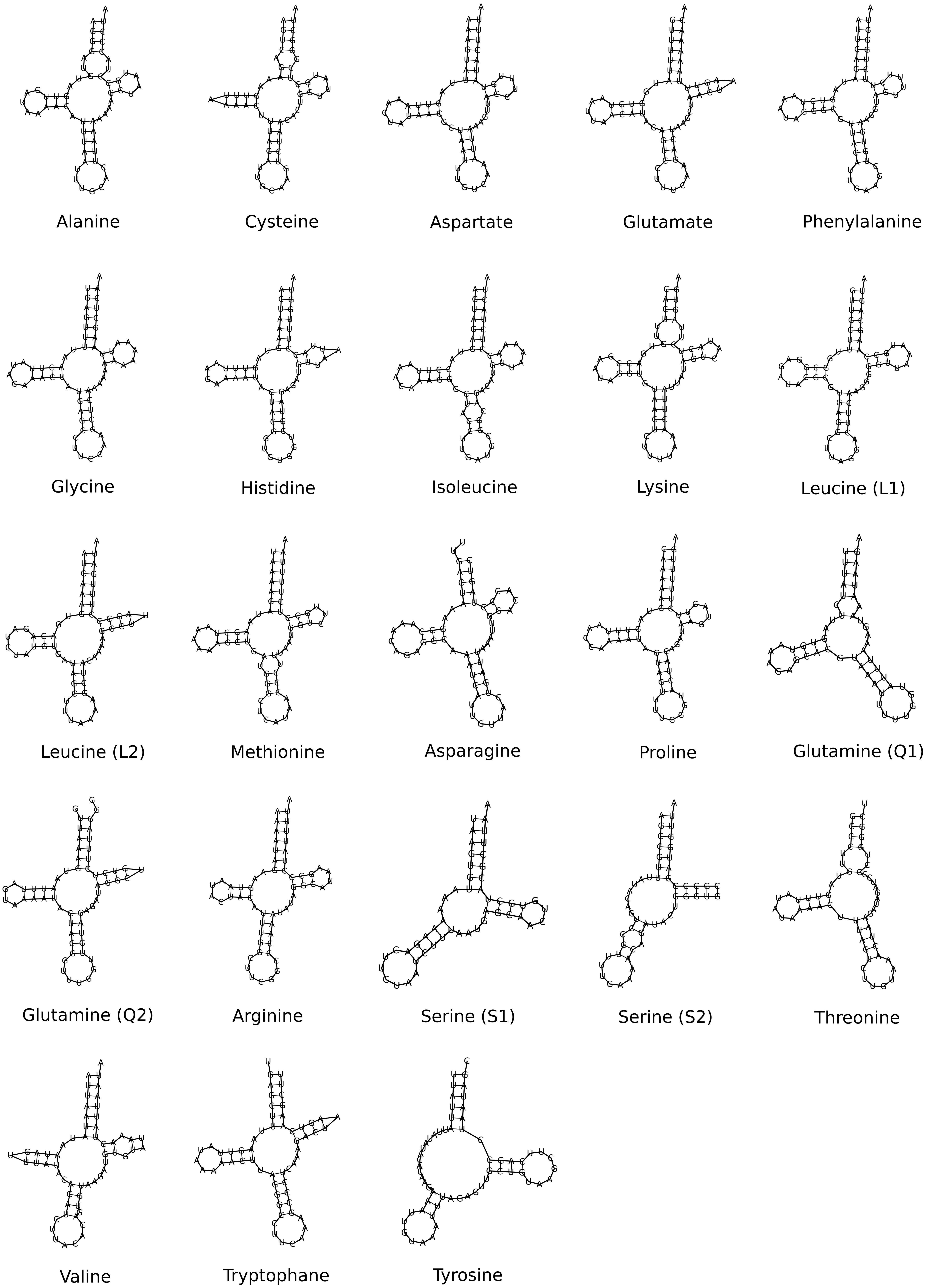

Linevichella vortex

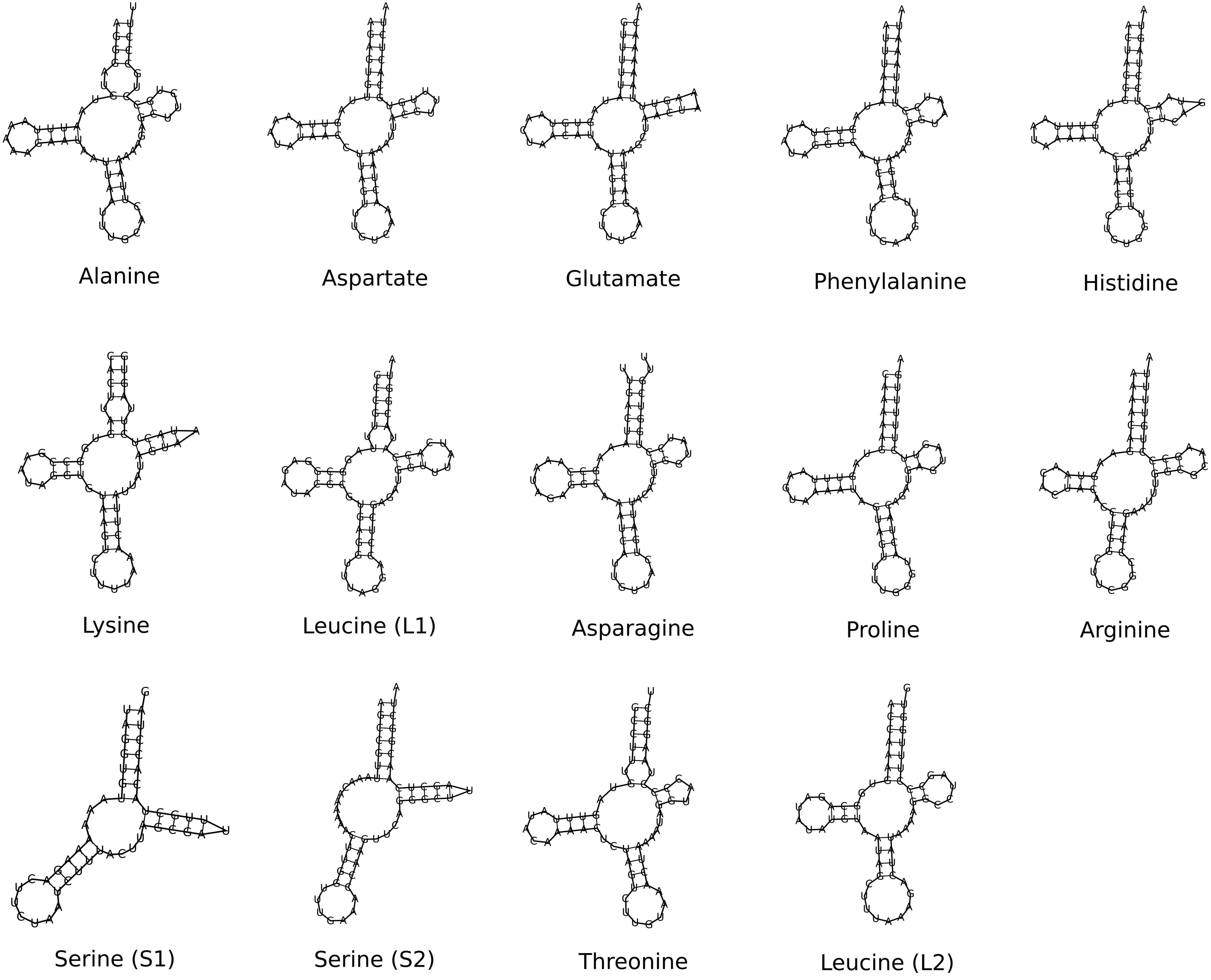

Pallaseopsis kesslerii

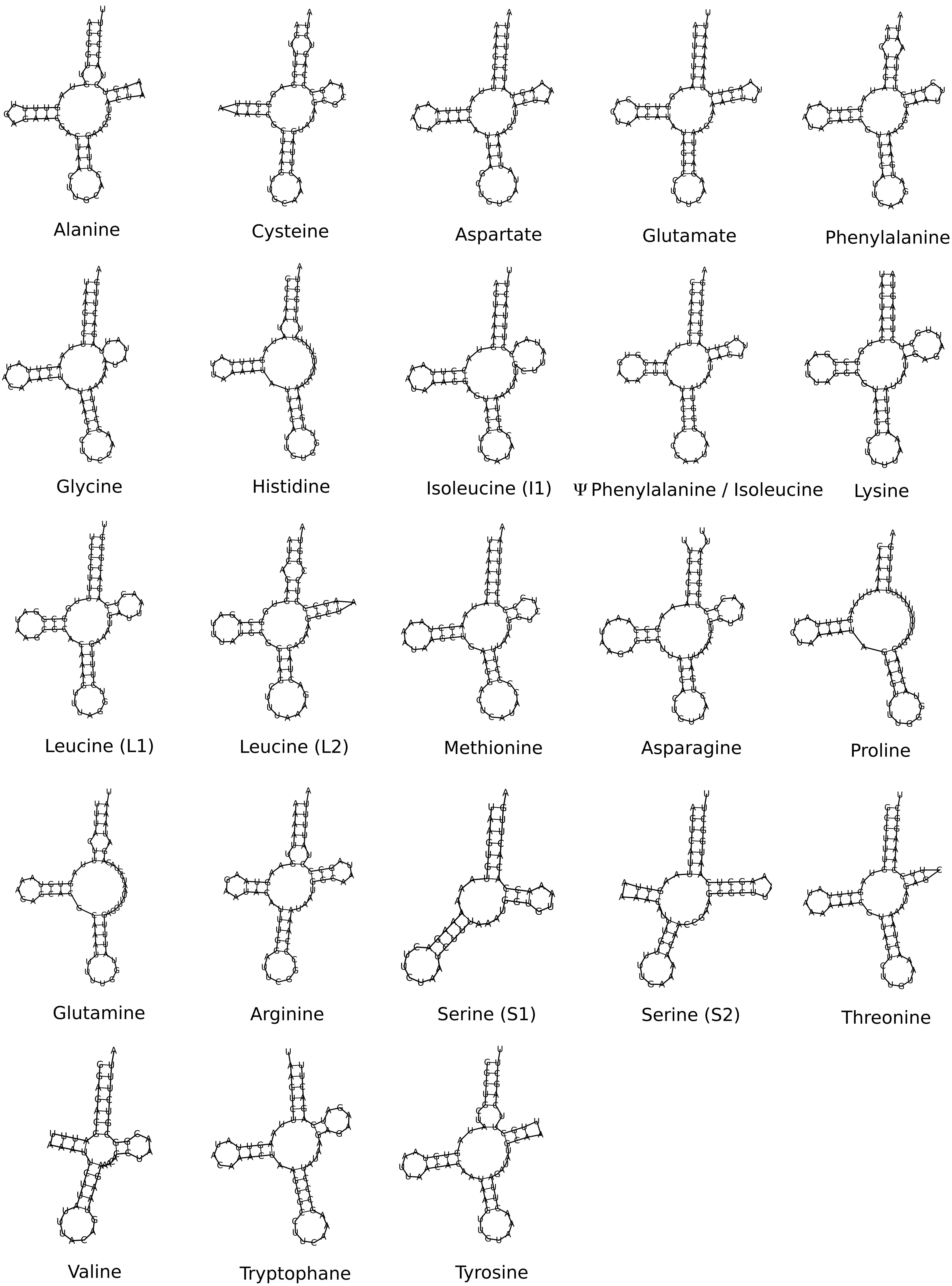

Supplement: Additional file 6: — The predicted mitochondrial tRNAs secondary structures of Baikalian amphipods under study. (PDF 9263 kb) [file 12864_2016_3357_MOESM6_ESM.pdf]
