# Supplementary figures and images for: Evolution of mitochondrial genomes in Baikalian amphipods
Source: BMC Genomics. 2016 Dec 28;17(Suppl 14):1016. doi: 10.1186/s12864-016-3357-z (PMC5249044; doi:10.1186/s12864-016-3357-z)

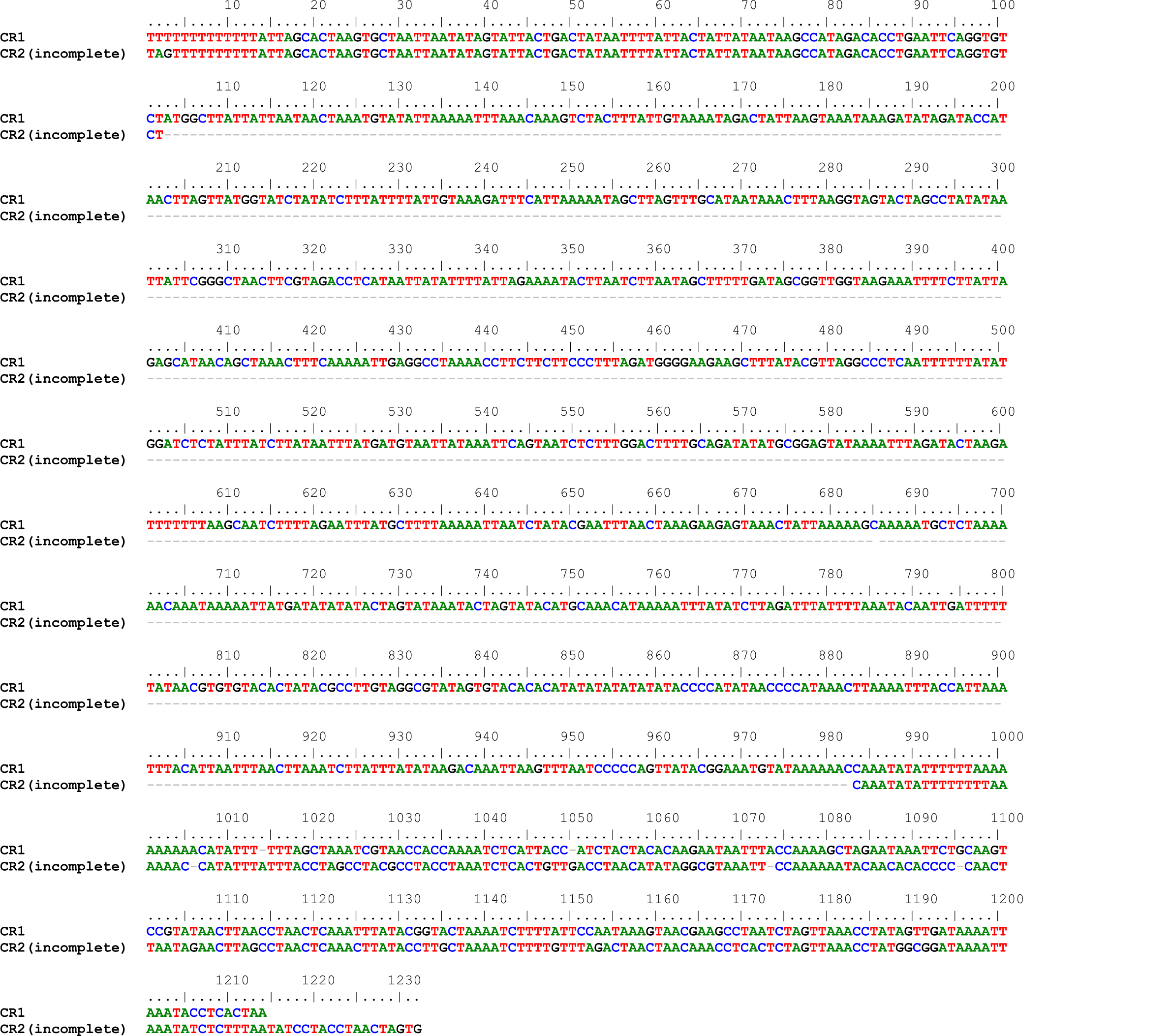

Supplement: Additional file 8: — Alignment of the putative CR sequence of Garjajewia cabanisii mitochondrial genome with its incompletely sequenced counterpart. Dashed lines indicate a missing sequence. (PNG 370 kb) [file 12864_2016_3357_MOESM8_ESM.png]

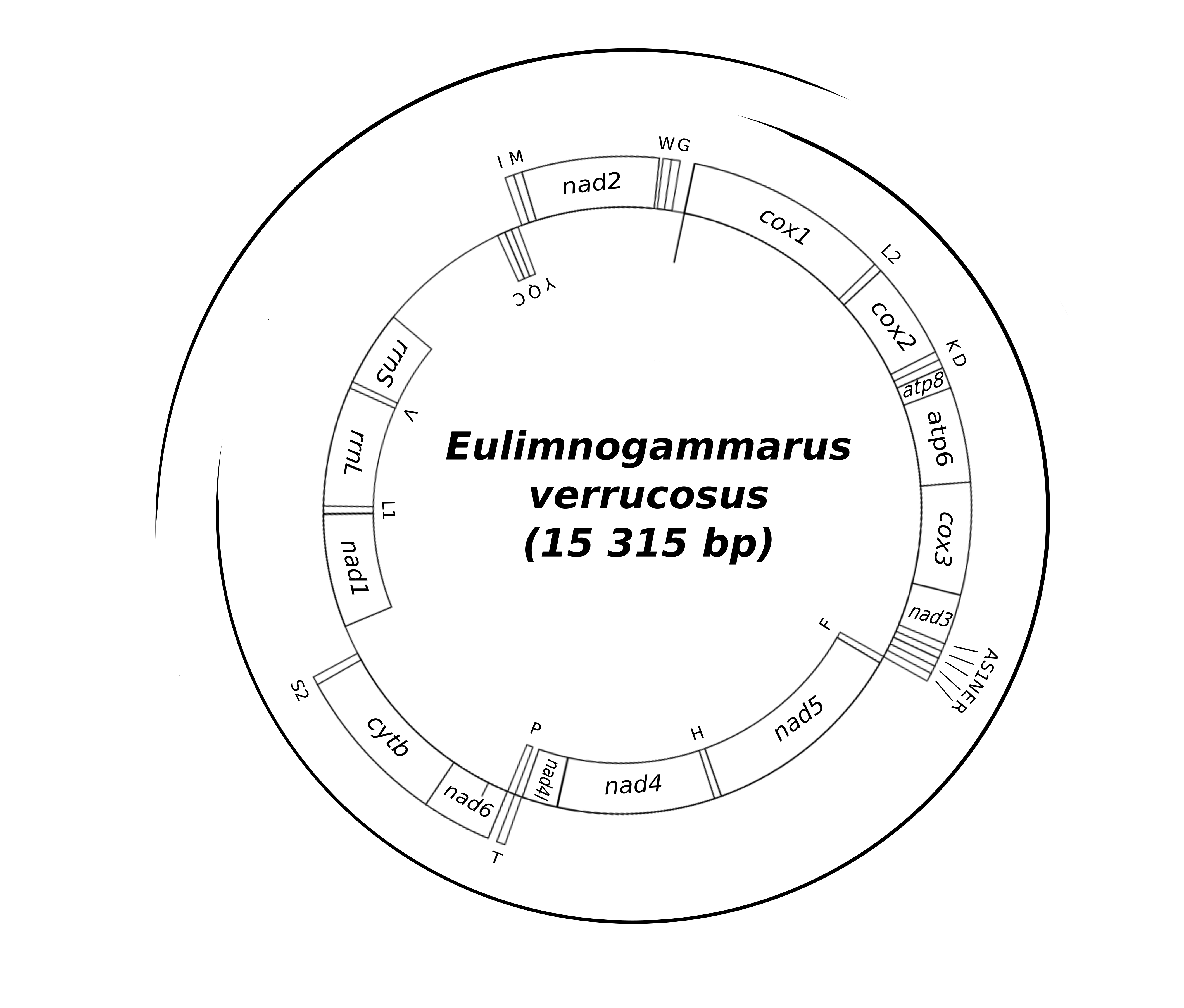

Supplement: Additional file 9: — The scheme of regions amplified by PCR in mitochondrial genomes of Baikalian amphipods. Eulimnogammarus verrucosus was used as an example of Baikalian amphipod. Protein-coding genes and ribosomal RNA genes are shown as sectors. Transfer RNA genes are labeled by their single-letter amino acid code. The features located on the (−) strand are shown inside the circle. The semicircles outside of the gene map denote the regions of the long-range amplification. (PNG 1167 kb) [file 12864_2016_3357_MOESM9_ESM.png]
